# Novel Polyomaviruses of Nonhuman Primates: Genetic and Serological Predictors for the Existence of Multiple Unknown Polyomaviruses within the Human Population

**DOI:** 10.1371/journal.ppat.1003429

**Published:** 2013-06-20

**Authors:** Nelly Scuda, Nadege Freda Madinda, Chantal Akoua-Koffi, Edgard Valerie Adjogoua, Diana Wevers, Jörg Hofmann, Kenneth N. Cameron, Siv Aina J. Leendertz, Emmanuel Couacy-Hymann, Martha Robbins, Christophe Boesch, Michael A. Jarvis, Ugo Moens, Lawrence Mugisha, Sébastien Calvignac-Spencer, Fabian H. Leendertz, Bernhard Ehlers

**Affiliations:** 1 Department of Infectious Diseases, Robert Koch Institute, Berlin, Germany; 2 Project 23 “Epidemiology of Highly Pathogenic Microorganisms,” Robert Koch Institute, Berlin, Germany; 3 Department of Primatology, Max Planck Institute, for Evolutionary Anthropology, Leipzig, Germany; 4 University Teaching Hospital Bouaké, Bouaké, Côte d'Ivoire; 5 Institut Pasteur Côte d'Ivoire, Abidjan, Côte d'Ivoire; 6 Institute of Virology, Charité - Universitätsmedizin Berlin, Berlin, Germany; 7 Mountain Gorilla Veterinary Project, Inc., Maryland, Baltimore, Maryland, United States of America; 8 LANADA/Laboratoire Central de la Pathologie Animale, Bingerville, Côte d'Ivoire; 9 School of Biomedical & Biological Sciences, University of Plymouth, Plymouth, United Kingdom; 10 University of Tromsø, Faculty of Health Sciences, Department of Medical Biology, Tromsø, Norway; 11 EcoHealth Research Group, Conservation & Ecosystem Health Alliance (CEHA), Kampala, Uganda; Washington University, United States of America

## Abstract

Polyomaviruses are a family of small non-enveloped DNA viruses that encode oncogenes and have been associated, to greater or lesser extent, with human disease and cancer. Currently, twelve polyomaviruses are known to circulate within the human population. To further examine the diversity of human polyomaviruses, we have utilized a combinatorial approach comprised of initial degenerate primer-based PCR identification and phylogenetic analysis of nonhuman primate (NHP) polyomavirus species, followed by polyomavirus-specific serological analysis of human sera. Using this approach we identified twenty novel NHP polyomaviruses: nine in great apes (six in chimpanzees, two in gorillas and one in orangutan), five in Old World monkeys and six in New World monkeys. Phylogenetic analysis indicated that only four of the nine chimpanzee polyomaviruses (six novel and three previously identified) had known close human counterparts. To determine whether the remaining chimpanzee polyomaviruses had potential human counterparts, the major viral capsid proteins (VP1) of four chimpanzee polyomaviruses were expressed in *E. coli* for use as antigens in enzyme-linked immunoassay (ELISA). Human serum/plasma samples from both Côte d'Ivoire and Germany showed frequent seropositivity for the four viruses. Antibody pre-adsorption-based ELISA excluded the possibility that reactivities resulted from binding to known human polyomaviruses. Together, these results support the existence of additional polyomaviruses circulating within the human population that are genetically and serologically related to existing chimpanzee polyomaviruses.

## Author Summary

Polyomaviruses are able to cause severe disease in immunocompromised individuals. The discovery of Merkel cell polyomavirus and its association with Merkel cell carcinoma has increased interest in these viruses, resulting in the identification of several novel human polyomaviruses in recent years. The existence of one of these recently identified viruses, human polyomavirus 9 (HPyV9), had been predicted nearly 30 years prior due to the ability of human sera to neutralize infection of an African green monkey polyomavirus (Lymphotropic polyomavirus; LPyV). HPyV9 and LPyV are now known to be antigenically and phylogenetically closely related. We hypothesized that nonhuman primate (NHP) polyomaviruses, in particular those of the closely related chimpanzee, may serve as genetic and immunological predictors for the existence of yet unknown human polyomaviruses. In the present study, we discovered 20 novel NHP polyomaviruses, six of which were isolated from chimpanzees. Of the 9 chimpanzee polyomaviruses now known, 5 do not presently have a closely related human counterpart. Serologic reactivity against these novel chimpanzee viruses was observed in humans from European and African populations. From these data we predict that additional human polyomaviruses exist which are genetically and serologically related to the novel chimpanzee polyomaviruses.

## Introduction

Over recent years the rate of identification of new viruses within human and animal populations has increased exponentially. Since 2007, more than 20 novel animal polyomaviruses have been discovered, and 12 genetically distinct human polyomaviruses are currently known. Polyomaviruses are non-enveloped viruses with a circular double-stranded DNA genome of approximately 5,000 base-pairs. All polyomaviruses encode proteins (large and small T antigens; LTag and STag) that have potential oncogenic capacity. However, transformation by these viruses is influenced by the individual virus type, as well as by the animal species undergoing infection [1]–[4]. With the exception of Merkel cell polyomavirus (MCPyV), the contribution of infection by polyomaviruses to human cancer remains unclear [5]–[7].

Infection with human polyomaviruses usually occurs in childhood or during adolescence without severe acute symptoms and results in lifelong persistence with no apparent disease. However, polyomavirus reactivation can cause serious disease in immunocompromised patients [8]. BK virus (BKPyV) was initially identified associated with nephropathy in renal transplant patients and with hemorrhagic cystitis in bone marrow transplant patients [9], [10]. Similarly, JCPyV was recognized as the causative agent of progressive multifocal leukoencephalopathy in iatrogenically immunosuppressed or HIV-infected individuals [11]. MCPyV was first identified in 2008, and has since been shown to be the etiological agent responsible for Merkel cell carcinoma [12]. Recently, a new human polyomavirus was detected in a patient suffering from *Trichodysplasia spinulosa*, and has been designated *Trichodysplasia spinulosa-*associated polyomavirus (TSPyV) [13]. Seven additional human polyomaviruses have been identified, but these viruses have not been linked to any disease [14]–[20]. Serological evidence indicates that most human adults have been exposed to many, if not all, of the known human polyomaviruses [20]–[25].

Human sera have been observed to serologically cross-react with polyomaviruses of nonhuman primates (NHPs) that have closely related human counterparts. For example, human sera reactive against BKPyV and JCPyV are cross-reactive with the closely related Old World monkey (OWM) polyomavirus simian virus 40 (SV40), and sera reactive against human polyomavirus 9 (HPyV9) have been shown to cross-react with the closely related OWM lymphotropic polyomavirus (LPyV) [21], [23], [25]–[27]. We propose that this cross-reactivity between human and closely related NHP polyomavirus counterparts may be used as an indicator for presence of unknown human polyomaviruses circulating within the human population.

In the present study, we have performed a comprehensive search for unknown NHP polyomaviruses by using degenerate primer-based PCR. Identified novel polyomaviruses were then sequenced to determine phylogenetic position within the polyomavirus family, followed by the use of serological assays of human sera to assess for the presence of reactivity against these newly identified NHP polyomaviruses. Our main focus was placed on chimpanzees, since they are our closest phylogenetic relatives and might therefore harbor polyomaviruses closely related to those found in humans [28]. We report on the discovery of 20 new NHP polyomaviruses (6 in chimpanzees), and the sequencing of 10 viruses at the complete genome level. Serological assays identify reactivity in human sera for a number of evolutionary distinct chimpanzee polyomaviruses, supporting the existence of currently unknown human polyomaviruses circulating within the human population.

## Results

### Identification and characterization of polyomaviruses in NHP

Degenerate primer PCR-based analysis was performed to ascertain the diversity of polyomaviruses in wild NHPs. For this analysis, blood, tissue and fecal samples (n = 792) collected from live or deceased great apes, OWMs, NWMs and prosimians (44 different species; Table S1) were analysed by using two generic polyomavirus PCRs (PCR1 and PCR2; Table S2). Both PCRs target highly conserved regions of the gene encoding for the major structural protein VP1, and had previously been successful in identifying multiple chimpanzee polyomaviruses [29], [30]. Testing of 359 samples with PCR1, and 433 samples with PCR2 identified 61/792 (8%) positive samples. Among the organs for which more than 20 samples were available, spleen, lymph node, intestine, and lung revealed the highest detection rates (20%, 16%, 15% and 7%, respectively). In addition, 3/7 skin samples (43%) were PCR-positive. In contrast, less than 4% of feces, blood, urine and kidney samples were PCR-positive (Table 1). The amplified VP1 sequences were shown by BLAST analysis to originate from 24 distinct polyomaviruses, all exhibiting less than 90% nucleic acid identity to each other, or to the corresponding region of known polyomaviruses. Novel polyomaviruses were detected in eight catarrhine (OWM and great apes) and four platyrrhine (NWM) species, and were provisionally named according to their host species as described in the Methods section.

**Table 1:** Sample material and polyomavirus content.

Sample material	No. of tested samples	No. (%) of polyomavirus-positive samples
Blood	139	2 (1)
Spleen	126	25 (20)
Feces	117	3 (3)
Lung	106	7 (7)
Kidney	70	0 (0)
Lymph node	63	10 (16)
Intestine	41	6 (15)
Liver	35	2 (6)
Chimp meal remain^a^	22	1 (5)
Urine	21	0 (0)
Muscle	17	3 (18)
Heart	11	0 (0)
Skin	7	3 (43)
Stomach	7	0 (0)
Thymus	5	0 (0)
Pancreas	3	0 (0)
Bone marrow	2	0 (0)
total	792	61 (8)

a Muscle or other tissues from red colobus monkeys partly consumed by chimpanzees.

We discovered 9 polyomaviruses in great ape species (six in chimpanzees; two in the gorilla; one in orangutan). These viruses were further characterized in the present study and are listed in Table 2. Four additional great ape polyomaviruses (three of chimpanzee; one of gorilla) showed a high similarity to the human MCPyV. Their full-genome sequence was previously published [30] (Table S3). Six chimpanzees were co-infected with combinations of multiple chimpanzee polyomaviruses. In five OWM and four NWM species we detected five and six novel polyomaviruses, respectively (Table 2). In one NWM (white-fronted capuchin; *Cebus albifrons*) co-infection was observed. Prosimian (strepsirrhine) polyomaviruses were not detected, which may be a reflection of the small (n = 20) sample size.

**Table 2:** Novel polyomaviruses detected in nonhuman primates.

Polyomavirus	Virus abbreviation	Host species	Origin of host^a^	PCR-positive tissue(s)	PCR-positive individuals
*Ateles paniscus* polyomavirus 1	ApanPyV1	Red-faced spider monkey	Germany (c)	Intestine, liver, lung, spleen	1
*Cebus albifrons* polyomavirus 1	CalbPyV1	White-fronted capuchin	Germany (c)	Lung, spleen	1
*Cebus albifrons* polyomavirus 2	CalbPyV2	White-fronted capuchin	Germany (c)	Spleen	1
*Cebus albifrons* polyomavirus 3	CalbPyV3	White-fronted capuchin	Germany (c)	Skin	1
*Cercopithecus erythrotis* polyomavirus 1	CeryPyV1	Red-eared guenon	Cameroon (w)	Intestine, spleen	1
*Gorilla beringei graueri* polyomavirus 1	GbergPyV1	Eastern gorilla	DRC^b^ (w)	Feces	1
*Gorilla gorilla gorilla* polyomavirus 2	GgorgPyV2	Western gorilla	Cameroon (w)	Lymph node	1
*Macaca fascicularis* polyomavirus 1	MfasPyV1	Crab-eating macaque	Germany (c)	Lymph node, spleen	2
*Pan troglodytes verus* polyomavirus 3	PtrovPyV3	Western chimpanzee	Côte d'Ivoire (w)	Feces, lymph node, spleen	1
*Pan troglodytes verus* polyomavirus 4	PtrovPyV4	Western chimpanzee	Côte d'Ivoire (w)	Spleen	1
*Pan troglodytes verus* polyomavirus 5	PtrovPyV5	Western chimpanzee	Côte d'Ivoire (w)	Feces, lung, lymph node, spleen	4
*Pan troglodytes verus* polyomavirus 6	PtrovPyV6	Western chimpanzee	Côte d'Ivoire (w)	Muscle, skin	1
*Pan troglodytes troglodytes* polyomavirus 1	PtrotPyV1	Central chimpanzee	Gabon (s)	Feces	1
*Pan troglodytes schweinfurthii* polyomavirus 2	PtrosPyV2	Eastern chimpanzee	Uganda (s)	Blood	1
*Piliocolobus badius* polyomavirus 1	PbadPyV1	Western red colobus	Côte d'Ivoire (w)	Lung	1
*Piliocolobus badius* polyomavirus 2	PbadPyV2	Western red colobus	Côte d'Ivoire (w)	Liver, lung, lymph node, spleen	4
*Piliocolobus rufomitratus* polyomavirus 1	PrufPyV1	Eastern red colobus	DRC (w)	Spleen	1
*Pithecia pithecia* polyomavirus 1	PpitPyV1	White-faced saki	Germany (c)	Blood, lung, lymph node, spleen	2
*Pongo pygmaeus* polyomavirus 1	PpygPyV1	Bornean orangutan	Germany (c)	Spleen	1
*Saimiri sciureus* polyomavirus 1	SsciPyV1	Squirrel monkey	Germany (c)	Lymph node, spleen	2

a Living conditions: w, wild; c, held in captivity; s, born in the wild but kept in a sanctuary.

b DRC = Democratic Republic of Congo.

To characterize the complete genomes of the nine great ape and 11 monkey polyomaviruses, specific nested primers in an inverse orientation were used to target the partial VP1 sequences obtained from the initial degenerate primer PCR for long-distance PCR amplification of the remaining virus genome. Complete genome sequences of ten polyomaviruses were amplified and sequenced: four from chimpanzees [Western chimpanzee (*Pan troglodytes verus*) and Eastern chimpanzee (*Pan troglodytes schweinfurthii*)], three from OWMs [Eastern red colobus (*Piliocolobus rufomitratus*), red-eared guenon (*Cercopithecus erythrotis*) and crab-eating macaque (*Macaca fascicularis*)] and three from NWMs [black spider monkey (*Ateles paniscus*), white-fronted capuchin (*Cebus albifrons*) and common squirrel monkey (*Saimiri sciureus*)]. Repeated amplification attempts from the remaining ten polyomaviruses were unsuccessful, most likely due to low genome copy numbers. The sequence information of these ten complete genomes and ten partial VP1 sequences has been deposited in the GenBank database. The accession numbers are listed in Table S3.

The full-length genomes have a length of 4970 bp to 5349 bp and exhibit the typical set of polyomavirus open reading frames (ORFs). The early regions are comprised of two ORFs encoding the non-structural proteins LTag and STag. The late regions code for the structural proteins VP1, VP2 and VP3, separated by a non-coding control region (NCCR). Only CeryPyV1 (from red-eared guenon) also harbors sequence information for a putative agnoprotein ORF. An ORF encoding a middle T-antigen was not identified in any of the viruses. The ORF locations and their lengths are listed in Table S4.

We examined the NCCRs for the presence of LTag binding sites (GAGGC) and inverted repeats (see Text S1; Figures S1 and S2). We also performed a detailed analysis of LTag for sequences corresponding to known functional motifs described in the SV40-derived form of the protein (see Text S2; Figures S3, S4, and S5; Table S5). All NCCRs possess one or several LTag binding sites and AT-rich stretches. Only the MfasPyV1 NCCR contains an inverted repeat (Figure S1). The LTag of all 10 novel nonhuman primate polyomaviruses contain a K/R-rich nuclear localization signal and CR1, DnaJ, Zn-finger and ATPase consensus motifs. Remarkably, only 6 out of the 10 possess the conserved LXCXE pRb1 pocket, suggesting some of the LTag of these novel polyomaviruses may not bind the retinoblastoma protein. Putative interaction domains with Bub-1 and CUL-7 are present in some of the LTag (Table S5; Figures S3 and S4).

### Phylogenetic analysis of the novel NHP polyomaviruses

**Figure 1:**
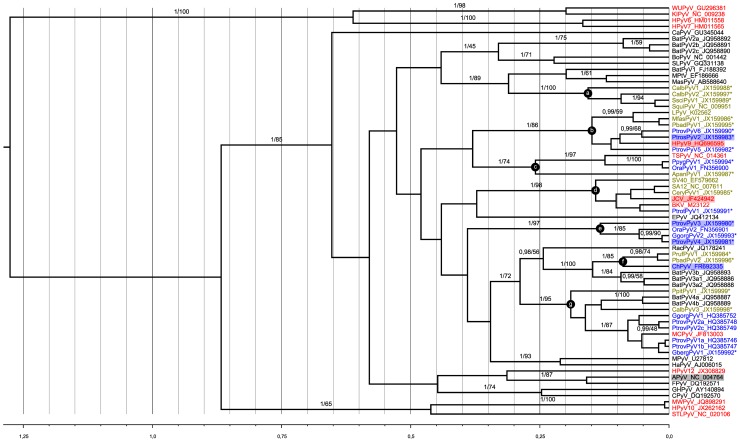
Bayesian chronogram deduced from the analysis of a 244 amino acid alignment of VP1 sequences. Polyomaviruses were identified in humans (red), apes (blue), other primates (green), and other mammals and birds (black). Novel polyomaviruses identified in this study are marked with a star and relevant clades to which they belong are highlighted by lettered circles. Viruses from which VP1 was used in serological assays are highlighted by colored rectangles. The human polyomavirus MXPyV has the same phylogenetic position as HPyV10 and is not shown. Support values are given above branches where posterior probability (pp) >0.95 and bootstrap values (Bp) >50. The tree presented is the maximum clade credibility tree. The scale axis is indicated in amino acid substitutions per site.

Ancient recombination events among polyomavirus lineages has recently been evidenced [31]. We therefore conducted independent phylogenetic analyses on amino acid alignments of three coding regions, VP1, VP2 and LTag (respective alignment lengths: 244, 90 and 443 amino acids). All alignments were comprised from the novel polyomaviruses and those currently available in GenBank, including all known human polyomaviruses (as of February 2013; Table S3). Maximum likelihood and Bayesian analyses of these alignments were performed. This confirmed the likely recombinant nature of some polyomaviruses and notably of those belonging to the *Wukipolyomavirus* genus (Figure 1; Figures S6 and S7). In addition, it also revealed that primate polyomaviruses were scattered over the entire polyomavirus tree, whether considering VP1, VP2 or large T phylogenetic trees (Figure 1; Figures S6 and S7). We identified 7 well-supported clades relevant to the novel polyomaviruses described in this study (Figure 1; Supplemental Figures S6 and S7; Table 3):

**Table 3:** Branch support values for selected clades in VP1, VP2 and large T phylogenetic analyses.

Clade	VP1	VP2	Large T
a^a^	1/100^b^	-/-c	1/100
B	1/86	1/98	1/100
C	1/74	1/97	1/100
D	1/98	1/89	1/100
E	1/97	1/92	1/100
F	1/85	1/90	1/94
G	1/95	na^d^	na

a Clades are designated by the same letter code as used in Figure 1.

b Branch support values are given as posterior probabilities/bootstrap values. The corresponding phylogenetic trees are available as Figure 1 (VP1), Figure S6 (VP2) and Figure S7 (large T).

c -: not a clade in the corresponding analysis.

d na: not applicable, i.e., none of the novel polyomaviruses included in group g allowed for whole genome recovery.

Clade (a) comprised four NWM polyomaviruses, CalbPyV1 and CalbPyV2 from white-fronted capuchin (*Cebus albifrons*), and SsciPyV1 and SqPyV1 from squirrel monkey (*Saimiri sciureus*).
Clade (b) consisted of three novel chimpanzee viruses [PtrovPyV5 and 6 from Western chimpanzees (*Pan troglodytes verus*) and PtrosPyV2 from Eastern chimpanzee (*Pan troglodytes schweinfurthii*)] and two novel viruses from cercopithecids [PbadPyV1 from Western red colobus (*Piliocolobus badius*), MfasPyV1 from crab-eating macaques (*Macaca fascicularis*)] which were associated to HPyV9 and LPyV.
Clade (c) included two orangutan viruses (PpygPyV1 and OraPyV1), a NWM polyomavirus [ApanPyV1 from red-faced spider monkey (*Ateles paniscus*)] and the human-infecting TSPyV.
Clade (d) comprised a chimpanzee and a monkey polyomavirus [PtrotPyV1 from Central chimpanzee (*Pan troglodytes troglodytes*) and CeryPyV1 from red-eared guenon (*Cercopithecus erythrotis*)] which formed a cluster with JCPyV, BKPyV, SV40 (from rhesus monkey) and SA12 (from baboon).
Clade (e) was constituted of four great ape polyomaviruses, PtrovPyV3 and PtrovPyV4 from Western chimpanzees, GgorgPyV2 from Western lowland gorilla (*Gorilla gorilla gorilla*) and OraPyV2 from Sumatran orangutan.
Clade (f) included two colobus viruses [PrufPyV1 from Eastern red colobus (*Piliocolobus rufomitratus*) and PbadPyV2 from Western red colobus] and the chimpanzee virus ChPyV.
In clade (g) finally, GbergPyV1 from Eastern lowland gorilla (*Gorilla gorilla beringei*), grouped with MCPyV and MCPyV-related great ape polyomaviruses. These viruses were associated to two polyomaviruses detected in NWMs [CalbPyV3 from white-fronted capuchin and PpitPyV1 from white-faced saki (*Pithecia pithecia*)], as well as to some bat polyomaviruses.

### Reactivity of human sera against VP1 of chimpanzee polyomaviruses

**Figure 2:**
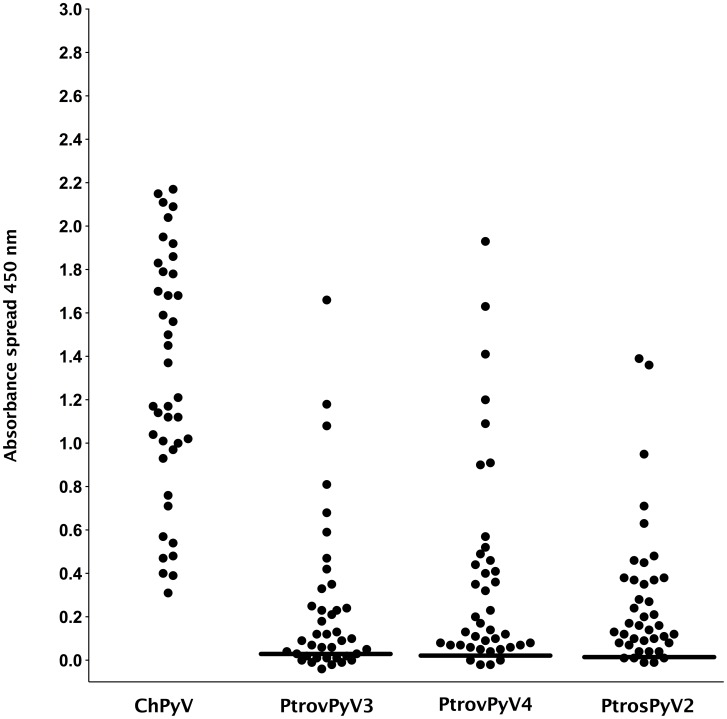
Reactivity of chimpanzee plasma samples to VP1 proteins of chimpanzee polyomaviruses. Antibody reactivity was assessed against the 4 chimpanzee polyomaviruses ChPyV, PtrovPyV3, PtrovPyV4 and PtrosPyV2 using plasma of 40 chimpanzees. Samples were analysed for seroreactivity with a capsomer-based IgG ELISA using the VP1 major capsid protein of the above polyomaviruses as antigens. The spread of absorbance measurement is shown with black dots, and cut-off values (COVs) are depicted with solid lines (PtrovPyV3: 0.028; PtrovPyV4: 0.023; PtrosPyV2: 0.013). A COV for ChPyV could not be calculated because all OD_450_ values were >0.3.

**Figure 3:**
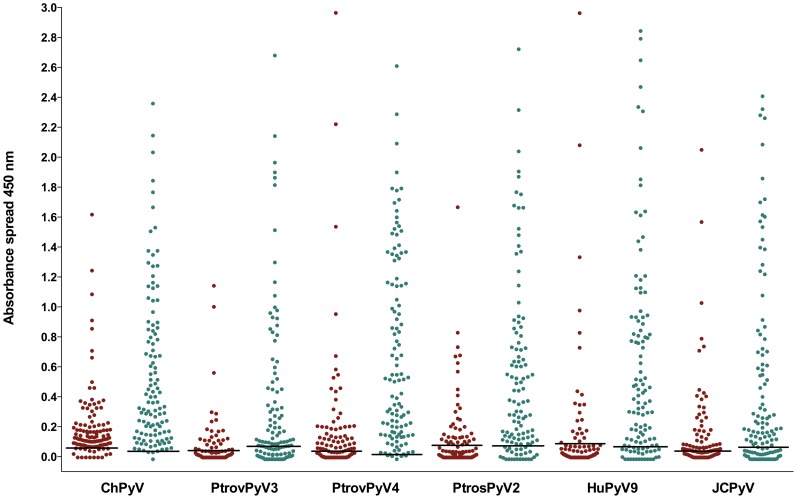
Reactivity of human sera to VP1 proteins of chimpanzee and human polyomaviruses. Antibody reactivity was assessed against 4 chimpanzee polyomaviruses (ChPyV, PtrovPyV3, PtrovPyV4 and PtrosPyV2) and 2 human polyomaviruses (HPyV9 and JCPyV) using sera from German (n = 111) and of plasma samples from Ivorian subjects (n = 115). Samples were analysed for seroreactivity with a capsomer-based IgG ELISA using the VP1 major capsid protein of the above polyomaviruses as antigens. The spread of absorbance measurement is shown with green and red dots (representing the German and Ivorian panels, respectively). COVs are shown as solid lines within the graph (COVs of Germans/Ivorians: ChPyV: 0.057/0.034; PtrovPyV3: 0.046/0.070; PtrovPyV4: 0.038/0.012; PtrosPyV2: 0.081/0.080; HPyV9: 0.089/0.066; JCPyV: 0.047/0.079).

To study the reactivity of human sera against the NHP polyomaviruses, VP1 proteins from four completely sequenced chimpanzee polyomaviruses (ChPyV, PtrovPyV3, PtrovPyV4, PtrosPyV2) with no close counterparts in humans were selected for use in indirect ELISA [clades (b), (e) and (f) in Figure 1]. For these studies, VP1 from JCPyV and HPyV9 were selected as positive control proteins, and an avian polyomavirus [APyV, also known as *Budgerigar* fledgling disease virus (*BFDV*)] [32] was chosen as negative control. VP1 proteins expressed in *E. coli* are known to form pentameric capsomer structures [33], and have proved effective for analysis of polyomavirus serology [21], [25], [34], [35]. To serologically assess the level of ChPyV, PtrovPyV3, PtrovPyV4 and PtrosPyV2 circulating in chimpanzees, ELISA was performed on plasma samples of 40 chimpanzees. A high seroprevalence was shown for each virus (ChPyV, 100%; PtroPyV3; 73%; PtrosPyV4, 90%; PtrosPyV2, 88%). These results indicate that all 4 polyomaviruses are hosted by chimpanzees, with ChPyV being the most prevalent (Figure 2). A serum panel from German individuals and a plasma panel from individuals from Côte d'Ivoire were then evaluated for their reactivity to the 4 chimpanzee polyomaviruses and to JCPyV and HPyV9. For the German sera (n = 111), the following seroreactivities were determined: ChPyV, 84%; PtrovPyV3, 24%; PtrovPyV4, 50%; PtrosPyV2, 33%; HPyV9, 21%; JCPyV, 42% (Table 4). Fourteen German sera (13%) exhibited seroreactivity against all four chimpanzee polyomaviruses, and 14 samples (13%) were completely negative (Figure S8A). The Côte d'Ivoire plasma samples (n = 115) showed more frequent reactivity: ChPyV, 97%; PtrovPyV3, 60%; PtrovPyV4, 96%; PtrosPyV2, 77%; HPyV9, 76%; JCPyV, 65% (Table 4). Each plasma reacted with at least one chimpanzee polyomavirus, with fifty-three (46%) samples being reactive against all four tested chimpanzee polyomaviruses (Figure S8B). Comparison of German and Côte d'Ivoire samples revealed that the seroprevalences were lower in the German samples (P<0.001 for all viruses; Table 4; Figure 3). This difference was also observed when analysis was age-restricted to individuals between 20 and 60 years (P = 0.004 for ChPyV, P<0.001 for all other viruses). Mean absorbance values were significantly lower in German samples for all viruses (P<0.001 for all viruses) (Figure 3). Age had no significant effect on sera/plasma absorbance values against any virus in either Germans or individuals from Côte d'Ivoire (P>0.05 for all viruses; Figure S9). To visualize possible correlations of seroreactivity against the tested VP1 antigens, OD_450_ values were plotted against each other, and correlation analysis was performed to check for statistical evidence of cross-reaction. Rank correlation showed only slight to moderate correlations (0.178 to 0.62), and for none of the antigen pairs was a correlation >0.5 measured for any serum/plasma panel (Table S6), indicating the absence of marked cross-reactions.

**Table 4:** Seroreactivity of German sera and Ivorian plasma samples against polyomaviruses by age group.

		No. of ELISA-positive sera/plasma samples (%)											
		ChPyV		PtrovPyV3		PtrovPyV4		PtrosPyV2		HuPyV9		JCPyV	
Age groups	No. in age groups	German	Ivorian	German	Ivorian	German	Ivorian	German	Ivorian	German	Ivorian	German	Ivorian
≤20 years	6/10^a^	5 (83)	10 (100)	2 (33)	5 (50)	4 (66)	10 (100)	3 (50)	7 (70)	2 (33)	6 (60)	5 (83)	6 (60)
21–40 years	83/41	67 (81)	39 (95)	21 (25)	20 (49)	40 (48)	40 (98)	27 (33)	31 (76)	14 (17)	31 (76)	34 (41)	30 (73)
41–60 years	22/41	21 (95)	40 (98)	4 (18)	26 (63)	11 (50)	37 (90)	7 (32)	35 (85)	7 (32)	35 (85)	8 (36)	26 (63)
>60 years	-/17	-	17 (100)	-	13 (76)	-	17 (100)	-	13 (76)	-	11 (65)	-	9 (53)
unknown age	-/6	-	6 (100)	-	5 (83)	-	6 (100)	-	3 (50)	-	4 (66)	-	4 (66)

a number of sera from Germany before, number of plasma samples from Côte d'Ivoire after the slash;

-, not available.

### Cross-reactivity between chimpanzee and human polyomaviruses

To assess possible antigenic cross-reactivity between the four chimpanzee polyomaviruses and known human polyomaviruses, competitive inhibition of seroreactivity was tested. Serum and plasma samples (n = 5–7) reactive against VP1 of a particular chimpanzee polyomavirus (ChPyV, PtrovPyV3, PtrovPyV4 or PtrosPyV2) were tested by ELISA using the respective chimpanzee polyomavirus VP1 as the antigen. Prior to use in the assay, all sera were pre-adsorbed with soluble VP1 antigen from BKPyV, HPyV9, JCPyV, MCPyV or TSPyV. Incubation with the soluble homologous chimpanzee polyomavirus VP1, and soluble APyV VP1 served as positive and negative controls, respectively. Pre-adsorption with the homologous chimpanzee polyomavirus antigens reduced the ELISA reactivity by approximately 80% or more in all cases (Table 5), and pre-incubation with APyV VP1 had no effect on reactivity (data not shown). This showed efficacy and specificity of the pre-adsorption procedure. With one exception, pre-incubation with VP1 from human polyomaviruses did not reduce reactivity of sera for VP1 of ChPyV, PtrovPyV3, PtrovPyV4 or PtrosPyV2. In the one exception, pre-incubation of PtrosPyV2-reactive human sera with soluble HPyV9-VP1 reduced the PtrosPyV2-specific ELISA reactivity by 31%, indicating a potential weak cross-reactivity (Table 5). This cross-reactivity was consistent with presence of these two viruses in sister phylogenetic clades (Figure 1), with their VP1 proteins showing 75% identity. For this relatively high level of identity cross-reactive antibodies have been detected [21], [25]. However, the non-adsorbable reactivity between PtrosPyV2 and HPyV9 (Table 5) implied – beside HPyV9 – the involvement of PtrosPyV2 and/or another unknown polyomavirus in the reactivity of human sera against PtrosPyV2. In summary, the presence of reactivity in human sera against VP1 from multiple NHP polyomaviruses with no currently known human homologue supports the presence of one or more unidentified human polyomaviruses phylogenetically related to each of these novel NHP viruses.

**Table 5:** Competitive inhibition of seroreactivity between human and chimpanzee polyomaviruses.

	Seroreactivity in ELISA (%)			
Competing antigen^a^	ChPyV	PtrovPyV3	PtrovPyV4	PtrosPyV2
APyV	99	96	100	99
BKPyV	100	100	100	98
HPyV9	97	86	100	69
JCPyV	95	100	100	96
MCPyV	89	100	100	98
TSPyV	94	100	100	88
ChPyV	14	nt	nt	Nt
PtrovPyV3	nt^b^	9	nt	Nt
PtrovPyV4	nt	nt	14	Nt
PtrosPyV2	nt	nt	nt	20

a Before ELISA, 2 µg/ml of VP1 antigen was used for pre-adsorption of antibodies from human sera.

b not tested.

## Discussion

In the present study, multiple, hitherto unknown, highly diverse polyomaviruses were detected in great apes and monkeys. These viruses were localized mainly to lymphoid organs, lungs and intestinal tissue (Tables 1 and 2; Table S1). In phylogenetic analysis using VP1, VP2 and LTag antigen protein sequences, four chimpanzee polyomaviruses (ChPyV, PtrovPyV3, PtrovPyV4, PtrosPyV2) showed no close relationship to any of the known human polyomaviruses, including the most recently discovered human polyomaviruses HPyV10, MWPyV, MXPyV, STLPyV and HPyV12 (Figure 1 and Figures S6 and S7, respectively). Positive ELISA reactivities against the VP1 structural proteins of these four chimpanzee polyomaviruses were observed in panels of human sera/plasma samples. Experiments involving competitive inhibition of seroreactivities with a panel of VP1 proteins from five human polyomaviruses ruled out the presence of cross-reactivity between the chimpanzee polyomaviruses and human polyomaviruses (except for a weak cross-reactivity between HPyV9 and PtrosPyV2) (Table 5). This was confirmed by the lack of any significant correlation of seroreactivity against the different polyomavirus VP1 proteins for any of the sera/plasma samples tested. Therefore, the reactivity of human sera against the four chimpanzee polyomaviruses suggests that the majority of human subjects tested have been exposed to as yet unknown polyomaviruses. The use of serology for the detection of unknown polyomaviruses circulating within the human population is not without precedent. Several research groups had observed that up to 30% of human sera react against the monkey polyomavirus LPyV [21], [36], [37]. About 30 years after the first observation, it was discovered that human seroreactivity against LPyV was due to infection by HPyV9 [25], a human polyomavirus closely related to LPyV.

Ivorian plasma samples consistently showed higher levels of VP1 reactivity compared to samples from German individuals (Figure 3). One possible interpretation of this stronger reactivity is that it reflects increased ‘spillover’ of NHP polyomaviruses into humans, perhaps due to the possibility for closer interaction between humans and NHP species. However, the Ivorian samples reacted more strongly with all polyomaviruses investigated, including VP1 from the two human viruses, JCPyV and HPyV9. This observation indicates a generally higher sero-reactivity, and is most likely not a result of zoonotic transmission events. Instead, it may reflect African-European differences in humoral immunity, similar to the differences in cellular immunity observed previously between patients from Gabon and Austria [38], [39], and Cameroonese children compared to other African and Caucasian populations [39]. Such immunological differences, together with differences in the level of transmissibility of local viral strains as well as social factors influencing person-to-person transmission, may result in pronounced geographic differences in seroprevalence rates. Seroprevalences of the human polyomaviruses BKPyV and MCPyV have for example been shown to range from 25% to 100%, depending on the geographic origin of the samples [16], [21]–[23], [26], [27], [36], [40], [41].

Using degenerate PyV PCR in NHPs, we found a high prevalence of polyomaviruses in spleen, lymph node and intestine samples. This observation led us to test comparable human tissue samples for the presence of human counterparts of ChPyV, PtrovPyV3, PtrovPyV4 and PtrosPyV2. Surprisingly, human spleen and lymph node samples were largely PCR negative. However, we did identify a novel human polyomavirus in liver and intestine samples that showed no close genetic relationship to any of the known polyomaviruses (designated human polyomavirus 12; HPyV12) [20], and only exhibited 55%–62% amino acid identity with VP1 sequences of ChPyV, PtroPyV3, PtroPyV4 and PtrosPyV2. Cross-reactivity of VP1 proteins in serological assays have thus far only been observed for proteins of more than 75% identity [17], [21], [23], [24], [42], with polyomaviruses with lower VP1 identity values showing no cross-reactivity [42]–[44]. Therefore, we have substantial confidence that HPyV12 is not one of the putative unknown human polyomaviruses that were predicted in the present study.

Identifying the human polyomaviruses predicted in this study will likely be no easy task. Their lack of detection in the face of the massive screening effort performed by the scientific community over recent years already testifies that these viruses are not easy targets. The underlying reason could be technical. For example, although efficient generic PCR methods are available, there is no guarantee that the systems in use can amplify these elusive human polyomaviruses. Another explanation may also lie in the biology of these polyomaviruses. For example, their tissue tropism may hamper detection if the corresponding tissue type is not commonly used for polyomavirus detection and/or is difficult to obtain. Therefore, the gain of molecular information about these human polyomaviruses may require the use of alternative detection methods, e.g. PCR systems specifically designed to target meaningful subsets of polyomaviruses, and/or targeting of body compartments that have not commonly been analyzed. Importantly, the results from the present study can be used to develop targeted nucleic-acid based detection methods for their identification in the future. Clearly, the limitation of the serological approach is the inability to discern single from multiple polyomaviruses within a phylogenetically related group. However, this strategy does indicate the presence of at least one, if not multiple, human polyomaviruses closely related to ChPyV, PtrovPyV3 and PtrovPyV4 (and possibly PtrosPyV2), circulating at substantial levels within the human population. The specific identity of the human correlate polyomaviruses and the disease implications associated with infection by these viruses remain to be determined.

## Materials and Methods

### Ethic statement

General permission for sample collection from deceased wild primates was obtained from the authorities of national parks of each country. Deceased animals were found during the course of a long term project focused on the behavior and infectious disease in wild-living nonhuman primates, mainly in Côte d'Ivoire. Most animals included in this study had died due to anthrax and respiratory diseases [45], [46]. No animal was anaesthetised or handled for the sole purpose of sample collection.

All samples from sanctuary-living wild-born great apes were collected during routine health checks by the sanctuary on-site veterinarian. No animal was sampled specifically for this study, and diagnostics were performed at RKI at request by the respective sanctuary. Therefore, no approval from our institutional committee was needed. All samples were collected according to the guidelines: PASA 2004. Pan African Sanctuary Alliance Veterinary Manual. Available at http://www.panafricanprimates.org/.

For animals living in zoological gardens and primate facilities, samples were obtained during routine health checks by the zoo and facility veterinarians. No animal was sampled specifically for the present study. Therefore, no approval from our institutional committee was needed. All samples were collected according to the guidelines laid down by Fowler and Miller [47] and according to the rules of the respective zoological gardens and primate facilities. Samples collected during necropsies on primates which died from various causes in zoological gardens and primate facilities were also included in these studies.

For all samples, importations occurred according to German veterinary regulations for import of organic materials. Tissue and blood samples were exported with the appropriate CITES permissions from the respective country and Germany.

Plasma samples of human volunteers in Côte d'Ivoire were sampled under the permission of the ministry of health of Côte d'Ivoire and the Institute Pasteur Côte d'Ivoire. Written informed consent was obtained from all participants of the study. The study was performed in cooperation with local health professionals. The aim of the study (specifically, to study broadly zoonotic diseases in the region) was explained to the local population during various educational campaigns. German serum samples were anonymously collected ‘residual materials’, and the collection was approved by the ethics committee of the Charité - Universitätsmedizin Berlin. All samples were collected according to the declaration of Helsinki.

### Human serum and plasma collection

Plasma samples (n = 115) from Côte d'Ivoire were collected from 57 women and 58 men, (age range: 9–79 years; mean: 42 years; six samples without age information) participating in a broad study to investigate zoonotic diseases at the human – wildlife interface in Côte d'Ivoire. Serum samples (n = 111) were collected from healthy German adults (55 female/56 male, age range: 20–60 years; mean: 32 years) at the Charité University Hospital, Berlin, Germany.

### NHP sample collection and processing

A total of 792 blood, fecal and tissue samples were collected from live or deceased individuals of 44 primate species (apes, OWMs, NWMs and prosimians) [30], [48]. 316 samples originated from wild primates in Africa (n = 313) and South America (n = 3), 54 samples from wild-born great apes housed in wildlife sanctuaries in West and East Africa (n = 49) and Asia (n = 5). 422 samples derived from captive primates held in several zoological gardens and primate facilities in Europe. Protection measures for the collection of fecal samples and autopsies and extraction of DNA of blood and tissue samples as well as fecal samples were carried out as described previously [30]. Blood of 40 chimpanzees was collected in EDTA tubes living on Ngamba Island Chimpanzee Sanctuary, Uganda, between 2001 and 2008 during the annual routine health checks under anaesthesia. Plasma was separated by centrifugation at 3000 rpm for 10 minutes at room temperature.

### Amplification and sequencing of polyomavirus genome sequences

Two generic PCRs for polyomavirus identification [29], [30] and long-distance PCR for genome amplification, as well as PCR product purification and sequencing were carried out as described previously [30]. For each novel polyomavirus nested specific primers for long-distance PCR were derived from the sequences amplified with the generic PCR. The primer pairs are listed with their annealing temperatures in Table S2.

### Sequence analysis

Complete/partial VP1, VP2 and LTag protein coding sequences generated for this study were translated into amino acids using SeaView [49] before being assembled with representative sequences of all polyomaviruses currently recognized as species by the International Committee on Taxonomy of Viruses (ICTV [32] or possibly qualifying as new species according to recent publications.

The three sets of sequences were aligned with SeaView using Muscle [50] and on the T-Coffee webserver using T-Coffee [51], [52]. CORE indices were computed for all alignments using the T-Coffee webserver and the following command line: t_coffee -infile = filename -output = html -score. Average scores were comparable for the three protein alignments; Muscle alignments were used in the following. Well-aligned blocks were selected using Gblocks v0.91b [53] as implemented in SeaView, which resulted in retaining 90, 244 and 443 positions from the initial VP2, VP1 and LTag alignments.

Best-fit models of amino acid evolution were determined using ProtTest v3 [54]. The seven empirical matrices of substitution rates implemented in BEAST v1.7.4 [55] were assessed in combination with empirical or dataset-borne (+F) amino acid frequencies and various hypotheses of rate variation along sequences (rate heterogeneity, +G and/or proportion of invariant sites, +I). Likelihoods were computed for all resulting 56 models using the slow optimization option of ProtTest (parameter values, branch lengths and topology were optimized). Best-fit models were determined using a combination of statistics: Akaike information criterion (AIC), corrected AIC and three Bayesian information criteria (BIC). CpREV+ G was selected for VP2, WAG+I+G for VP1, WAG+I+G+F for LTag.

Phylogenetic analyses were then performed under the given models of amino acid evolution in ML and Bayesian frameworks. ML analyses were performed with PhyML v3.0 [56] as implemented on the PhyML webserver [57]. All analyses were performed using the BEST RANDOM option, meaning that one nearest-neighbor interchange (NNI) and one subtree pruning and re-grafting (SPR) search were started using a BIONJ tree while five additional SPR searches used random starting trees, the best of the seven resulting trees being chosen as the output. Where applicable, site-specific rate heterogeneity was modeled using a four-category gamma law (+G4). Branch lengths and topologies were optimized. Branch support was estimated by performing non-parametrical bootstrapping (Bp; 500 pseudo-replicates).

Bayesian analyses were performed using BEAST v1.7.4 and the associated suite of softwares [55]. For all analyses, a relaxed clock model was implemented so as to account for among lineage rate variation and a speciation model (birth-death model) was chosen as depicting the shape of the trees. Two Markov chain Monte Carlo (MCMC) runs of 10,000,000 generations were run under these conditions for each alignment, sampling trees and numerical values of model parameters every 1000 generations. Convergence of the runs was checked with Tracer v1.5 (available at http://tree.bio.ed.ac.uk/software/tracer/). Visual confirmation that the stationary distribution had effectively been reached was obtained for both runs (a plateau was observed). In addition, model parameters apparently converged to undistinguishable distributions for both runs. Finally, combined effective sample sizes (ESS) were above 200 for all parameters. Trees sampled after a visually conservative burn-in of 1,000,000 generations were assembled into a single file using LogCombiner v1.7.1 before the information that this tree sample (in total 20000 trees) contained was summarized onto the maximum clade credibility (MCC) tree with TreeAnnotator v1.7.4. Posterior probabilities (pp) were taken as branch support values.

All trees presented in this article were made up with FigTree v1.3.1 (available at http://tree.bio.ed.ac.uk/software/figtree/).

### Expression and purification of recombinant proteins

The sequences of the major capsid proteins VP1 of HPyV9, BKPyV, JCPyV, MCPyV, TSPyV, APyV, ChPyV, PtroPyV3, PtroPyV4 and PtrosPyV2 were codon-optimized, commercially synthesized (MrGene GmbH, Regensburg, Germany) and expressed in *E.coli* K12 as pentameric structures as described previously [25].

### Serological analysis

IgG ELISAs, including use of APyV VP1 as a negative control to exclude non-specific seroreactivity (due to binding of antibodies to conserved VP1 epitopes or due to unspecific binding), estimation of cut-off values, calculation of the correlation of antibody reactivity using the Spearman rank correlation test, and adsorption assays with soluble VP1 capsomers were performed essentially as described [25]. The only exceptions from the earlier cited protocol were dilution of serum and plasma samples 1∶100; and, in adsorption assays, serum and plasma samples were preincubated with 2 µg/ml of antigen.

### Statistical analyses

The database was established in Excel for Windows before being transferred into Stata (Stata/SE 10.0 for Windows, Stata Corp, College Station, TX) for statistical analyses. Absorbance values and prevalence of the individual viruses and the effect of age and gender on absorbance values were analyzed using regression models and Fischer exact test.

### Provisional nomenclature, abbreviations and nucleotide sequence accession numbers of novel nonhuman primate polyomaviruses

For the purpose of this paper, tentative names and abbreviations for the novel NHP polyomaviruses were derived from species and subspecies name of the host in which the virus was detected (for example *Pan troglodytes verus* polyomavirus, PtrovPyV) and listed in Table 2. Using this naming rationale, the MCPyV-related polyomaviruses of *Pan troglodytes verus*, *Pan troglodytes schweinfurthii* and *Gorilla gorilla gorilla*, published in our earlier study [30], were renamed for consistency. Old names: GggPyV, PtvPyV, PtsPyV; new names: GgorgPyV, PtrovPyV, PtrosPyV. Nucleotide sequence accession numbers of the novel NHP polyomaviruses are listed in Table S3.

## Supporting Information

Figure S1 **Large T antigen-binding sites in NCCRs of novel NHP polyomaviruses.** Sites are boxed.
(TIF)

Figure S2 **Sequence homology between NCCRs.** The NCCRs of (a) MfasPyV1 and PtrovPyV5 and (b) PtrosPyV2 and PtrovPyV5 were aligned. Identical nucleic acids are marked by vertical lines, gaps by hyphens.
(TIF)

Figure S3 **Alignment of the primary sequence of large T antigens and their functional motifs from novel NHP polyomaviruses.** The LTag proteins of all novel NHP polyomaviruses (with complete genomes amplified and sequenced) were aligned with the LTAg of SV40. Functional motifs are highlighted with different colors. The color code is shown below the alignment.
(TIF)

Figure S4 **Location of functional motifs in large T antigen.** LTag is represented by an open bar.
(TIF)

Figure S5 **Amino acid sequence identity between the host range domain of SV40 large T antigen and the C-terminal region of CeryPyV1 large T antigen.** Identical amino acids are highlighted in yellow.
(TIF)

Figure S6 **Bayesian chronogram deduced from the analysis of a 90 amino acid alignment of VP2 sequences.** Polyomaviruses were identified in humans (red), apes (blue), other primates (green), and other mammals and birds (black). Novel polyomaviruses identified in this study are marked with a star. Viruses from which VP1 was used in serological assays are highlighted by colored rectangles. Clades ‘a’ and ‘g’ (highlighted in Figure 1) are not highlighted in this figure as a consequence of the disruption of clade ‘a’ monophyly by BoPyV and the lack of sequence for any of the novel polyomaviruses associated to published ones within clade ‘g’. Support values are given above branches where posterior probability (pp) >0,95 and bootstrap values (Bp) >50. The tree presented is the maximum clade credibility tree. The scale axis is presented as amino acid substitutions per site.
(TIF)

Figure S7 **Bayesian chronogram deduced from the analysis of a 443 amino acid alignment of large T sequences.** Polyomaviruses were identified in humans (red), apes (blue), other primates (green), and other mammals and birds (black). Novel polyomaviruses identified in this study are marked with a star. Viruses from which VP1 was used in serological assays are highlighted by colored rectangles. Clade ‘g’ (highlighted in Figure 1) is not highlighted in this figure as a consequence of the lack of sequence for any of the novel polyomaviruses associated to published ones within clade ‘g’. Support values are given above branches where posterior probability (pp) >0.95 and bootstrap values (Bp) >50. The tree presented is the maximum clade credibility tree. The scale axis is presented as amino acid substitutions per site.
(TIF)

Figure S8 **Multiple seroreactivities against chimpanzee polyomaviruses in humans.** German sera (A) and Ivorian plasma samples (B) were tested for seroreactivity against ChPyV, PtrovPyV3, PtrovPyV4 and PtrovPyV10. The graph displays percentages of single and multiple reactivities.
(TIF)

Figure S9 **Age-stratified reactivity of human sera to VP1 proteins of chimpanzees and human polyomaviruses.** Antibody reactivity against 2 human polyomaviruses (HPyV9 and JCPyV) and 4 chimpanzee polyomaviruses (ChPyV, PtrovPyV3, PtrovPyV4 and PtrosPyV2) of sera from German (n = 111) and of plasma samples from Ivorian subjects (n = 115). Samples were analysed for seroreactivity with a capsomer-based IgG ELISA using the VP1 major capsid protein of the above polyomaviruses as antigens. Absorbance spread measurements are shown as blue dots, representing the German (left) and Ivorian panels (right), respectively. The COV is shown as dashed line (values are given in legend of Figure 3). Solid line within the graph: age trendline.
(TIF)

Table S1 **Primate species and tissues tested with generic polyomavirus PCR.**
(DOC)

Table S2 **Primers used for amplification of nonhuman primate polyomaviruses.**
(DOC)

Table S3 **Known and novel polyomaviruses used in phylogenetic analysis.**
(DOC)

Table S4 **Genomes and encoded proteins of the novel nonhuman primate polyomaviruses.**
(DOC)

Table S5 **Putative functional motifs in the large T-antigens of the novel NHP polyomaviruses.**
(DOCX)

Table S6 **Correlation of seroreactivities against VP1 antigens of polyomaviruses.**
(DOC)

Text S1 **LT-ag binding motifs in NCCR of novel NHP polyomaviruses.**
(DOCX)

Text S2 **Motifs in large T antigens of novel NHP polyomaviruse.**
(DOCX)
