# Supplementary figures and images for: Novel Polyomaviruses of Nonhuman Primates: Genetic and Serological Predictors for the Existence of Multiple Unknown Polyomaviruses within the Human Population
Source: PLoS Pathog. 2013 Jun 20;9(6):e1003429. doi: 10.1371/journal.ppat.1003429 (PMC3688531; doi:10.1371/journal.ppat.1003429)

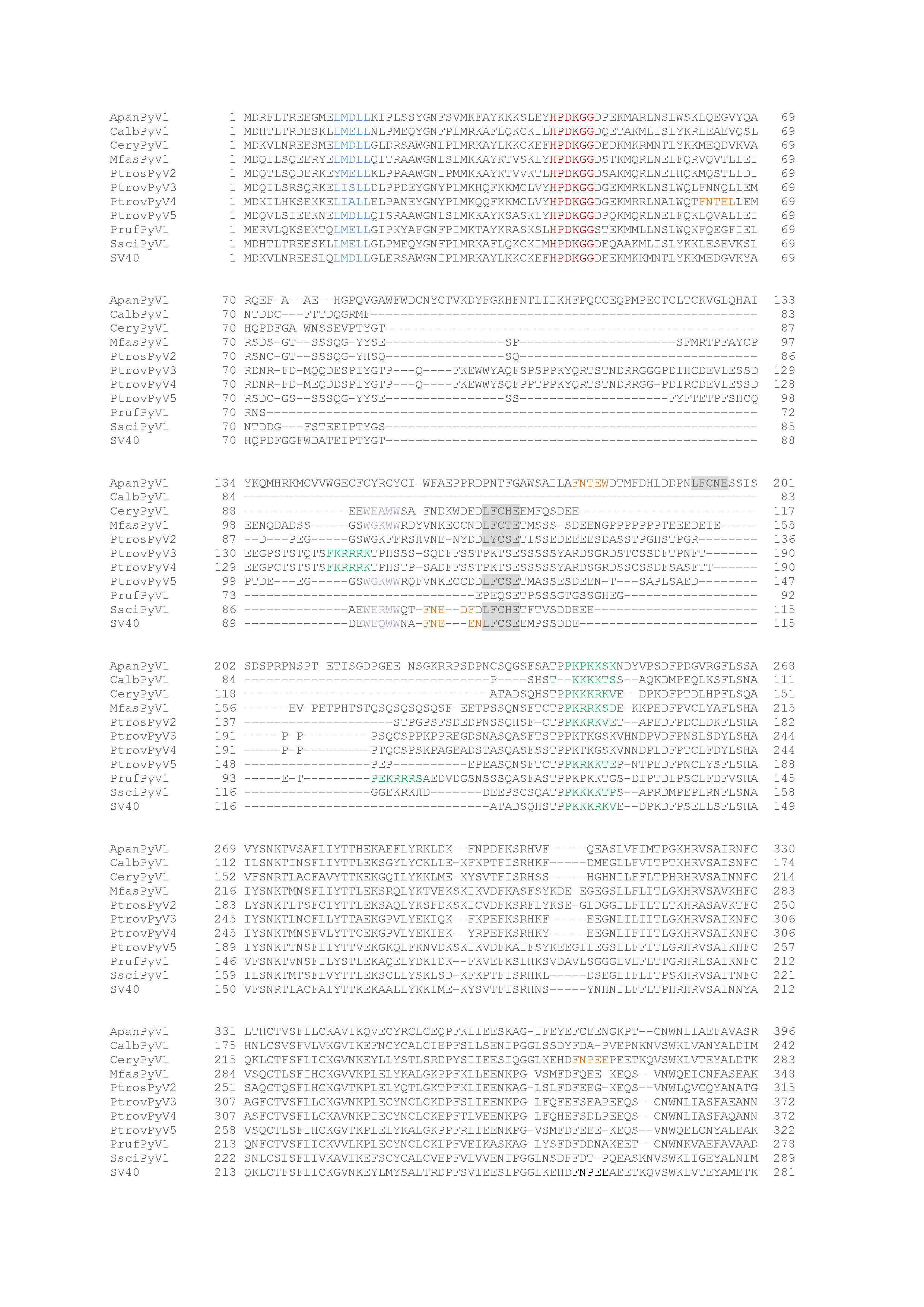

Supplement: Figure S3 — Alignment of the primary sequence of large T antigens and their functional motifs from novel NHP polyomaviruses. The LTag proteins of all novel NHP polyomaviruses (with complete genomes amplified and sequenced) were aligned with the LTAg of SV40. Functional motifs are highlighted with different colors. The color code is shown below the alignment. (TIF) [file ppat.1003429.s003.tif]

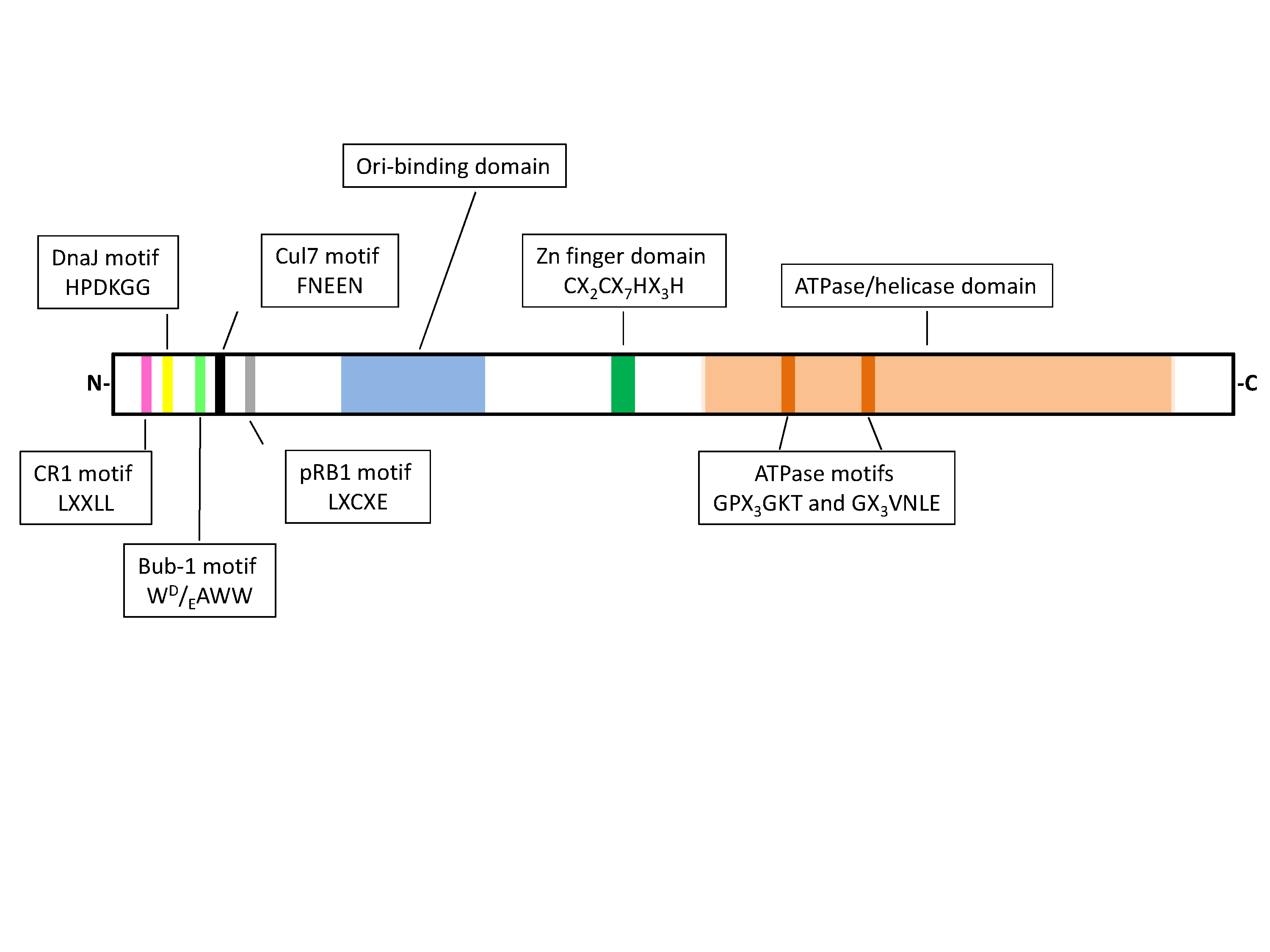

Supplement: Figure S4 — Location of functional motifs in large T antigen. LTag is represented by an open bar. (TIF) [file ppat.1003429.s004.tif]

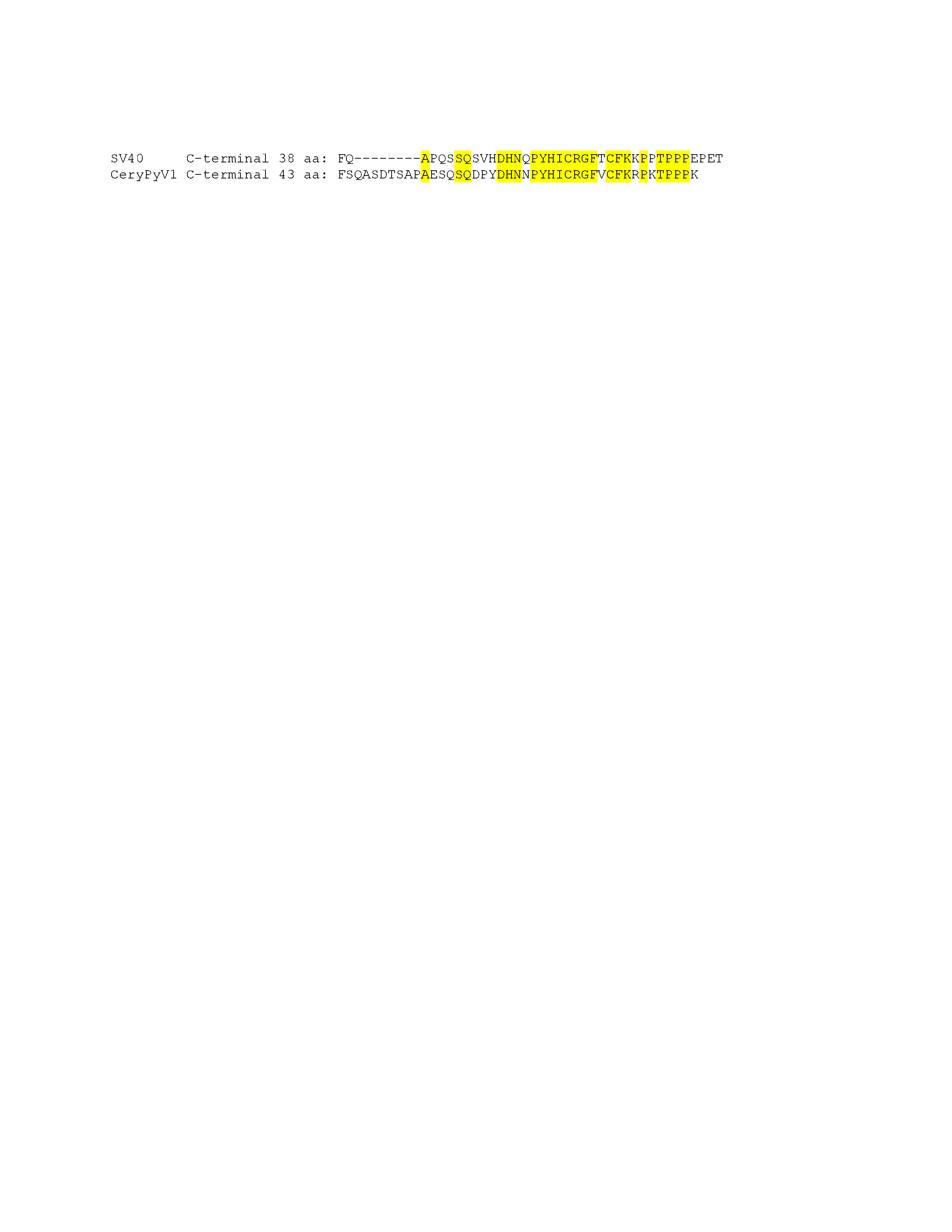

Supplement: Figure S5 — Amino acid sequence identity between the host range domain of SV40 large T antigen and the C-terminal region of CeryPyV1 large T antigen. Identical amino acids are highlighted in yellow. (TIF) [file ppat.1003429.s005.tif]

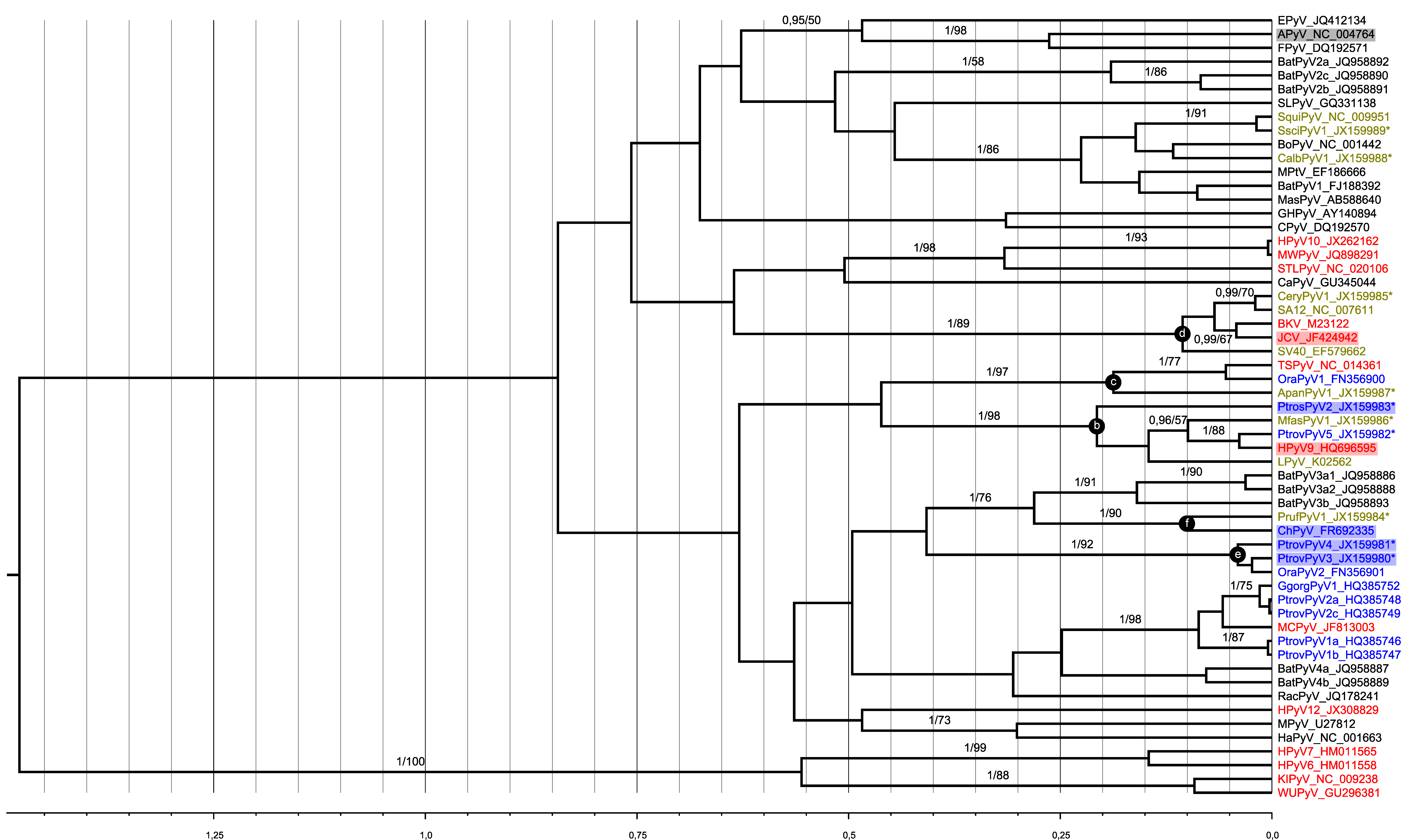

Supplement: Figure S6 — Bayesian chronogram deduced from the analysis of a 90 amino acid alignment of VP2 sequences. Polyomaviruses were identified in humans (red), apes (blue), other primates (green), and other mammals and birds (black). Novel polyomaviruses identified in this study are marked with a star. Viruses from which VP1 was used in serological assays are highlighted by colored rectangles. Clades ‘a’ and ‘g’ (highlighted in Figure 1) are not highlighted in this figure as a consequence of the disruption of clade ‘a’ monophyly by BoPyV and the lack of sequence for any of the novel polyomaviruses associated to published ones within clade ‘g’. Support values are given above branches where posterior probability (pp) >0,95 and bootstrap values (Bp) >50. The tree presented is the maximum clade credibility tree. The scale axis is presented as amino acid substitutions per site. (TIF) [file ppat.1003429.s006.tif]

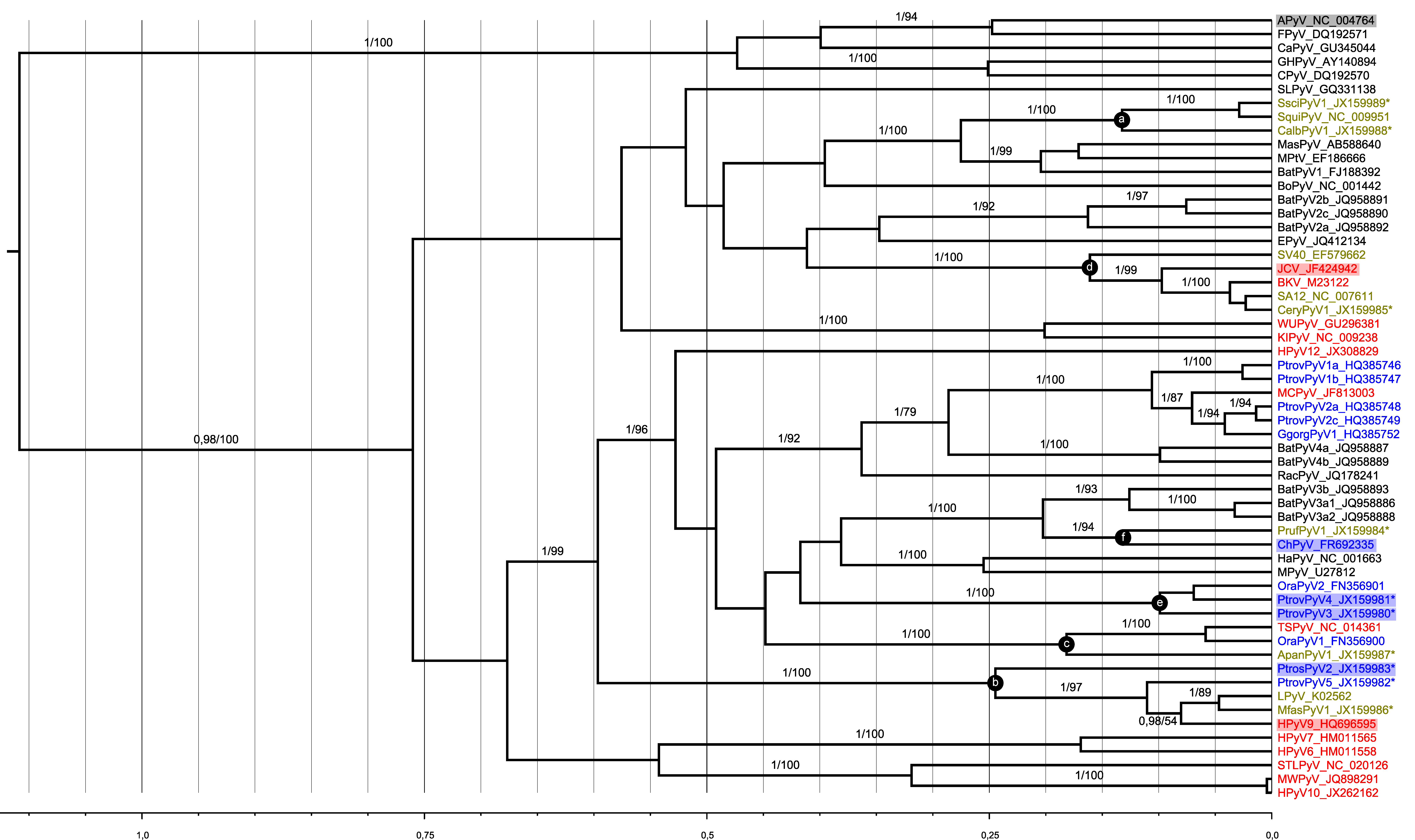

Supplement: Figure S7 — Bayesian chronogram deduced from the analysis of a 443 amino acid alignment of large T sequences. Polyomaviruses were identified in humans (red), apes (blue), other primates (green), and other mammals and birds (black). Novel polyomaviruses identified in this study are marked with a star. Viruses from which VP1 was used in serological assays are highlighted by colored rectangles. Clade ‘g’ (highlighted in Figure 1) is not highlighted in this figure as a consequence of the lack of sequence for any of the novel polyomaviruses associated to published ones within clade ‘g’. Support values are given above branches where posterior probability (pp) >0.95 and bootstrap values (Bp) >50. The tree presented is the maximum clade credibility tree. The scale axis is presented as amino acid substitutions per site. (TIF) [file ppat.1003429.s007.tif]

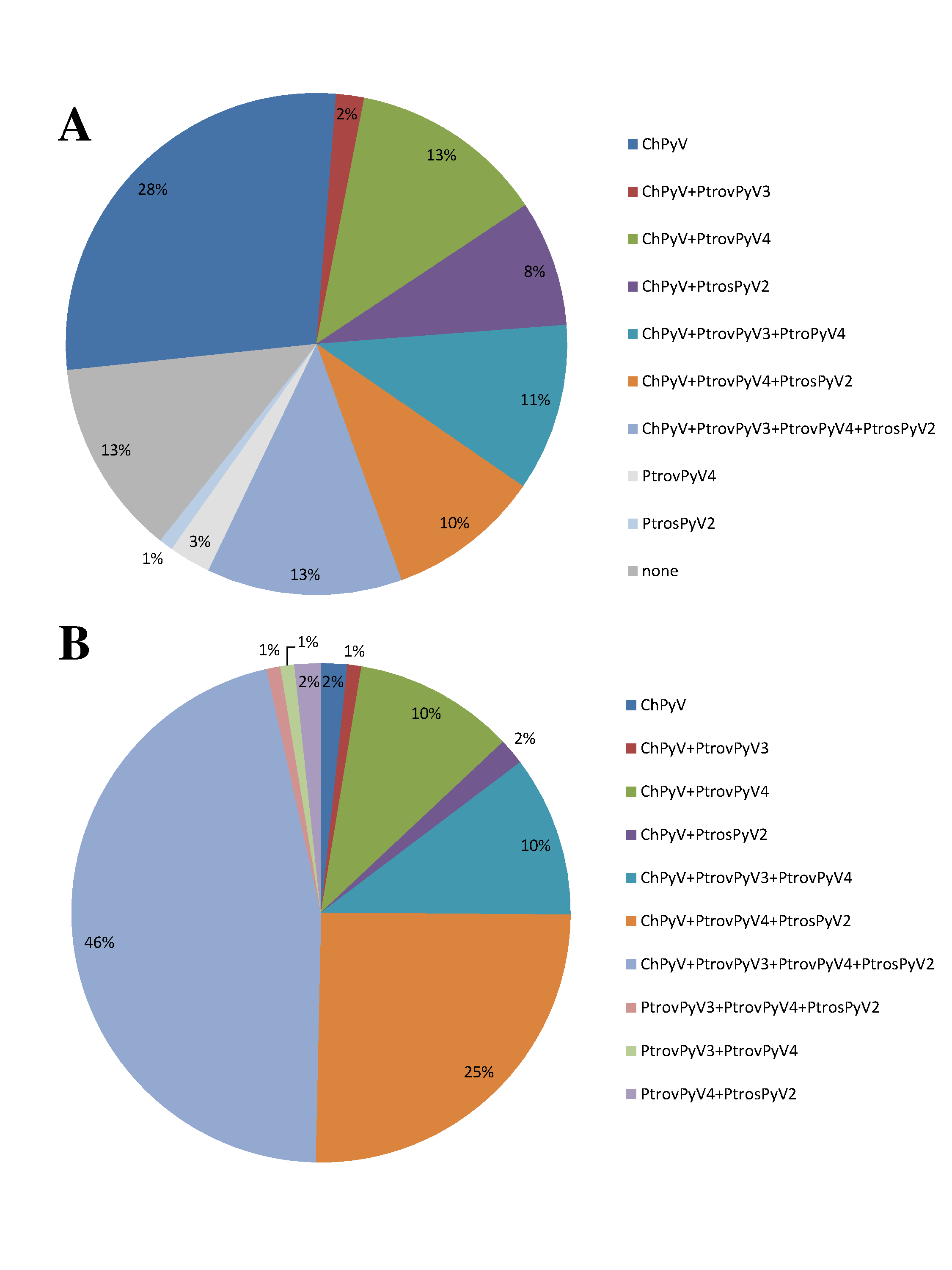

Supplement: Figure S8 — Multiple seroreactivities against chimpanzee polyomaviruses in humans. German sera (A) and Ivorian plasma samples (B) were tested for seroreactivity against ChPyV, PtrovPyV3, PtrovPyV4 and PtrovPyV10. The graph displays percentages of single and multiple reactivities. (TIF) [file ppat.1003429.s008.tif]

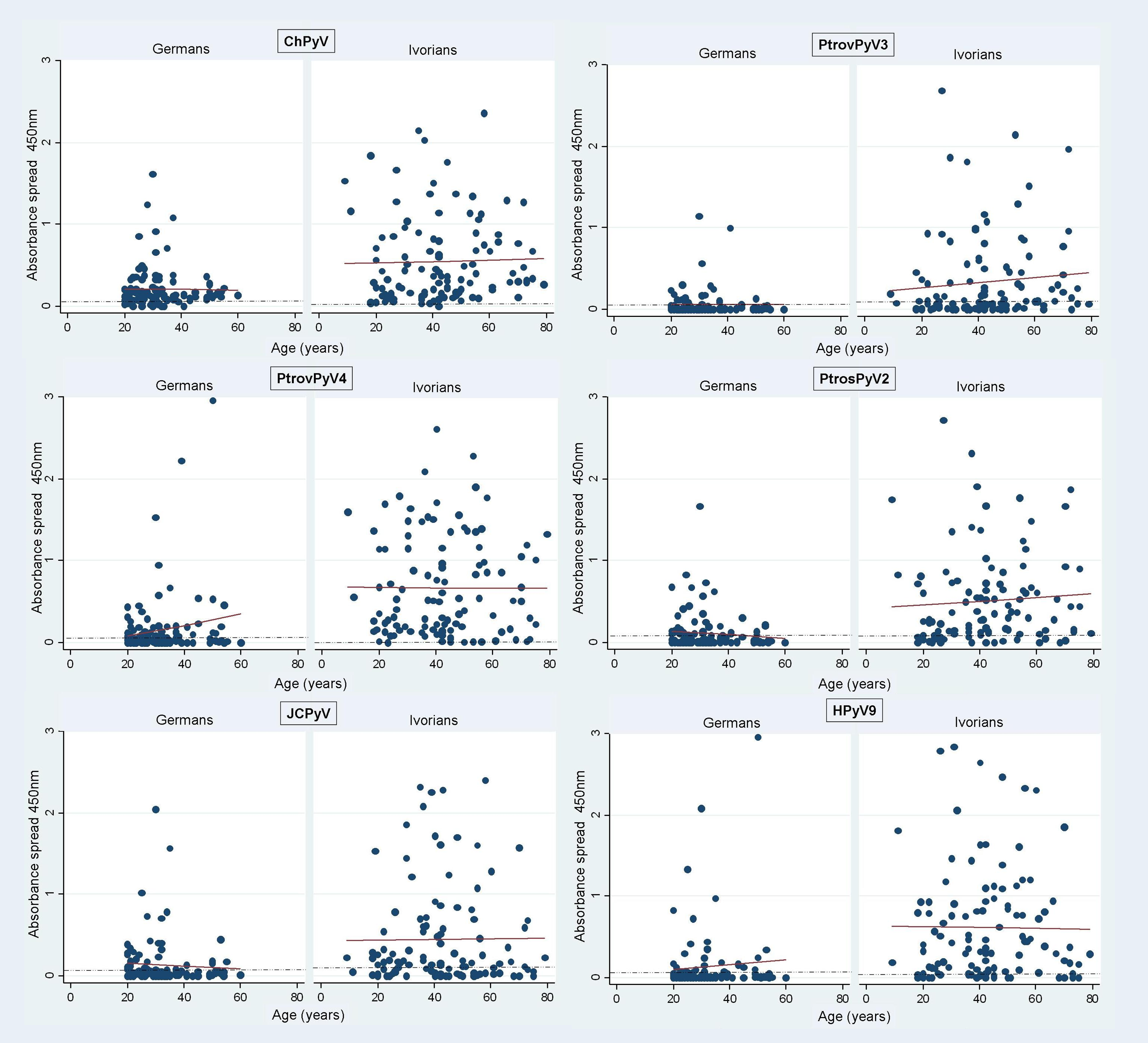

Supplement: Figure S9 — Age-stratified reactivity of human sera to VP1 proteins of chimpanzees and human polyomaviruses. Antibody reactivity against 2 human polyomaviruses (HPyV9 and JCPyV) and 4 chimpanzee polyomaviruses (ChPyV, PtrovPyV3, PtrovPyV4 and PtrosPyV2) of sera from German (n = 111) and of plasma samples from Ivorian subjects (n = 115). Samples were analysed for seroreactivity with a capsomer-based IgG ELISA using the VP1 major capsid protein of the above polyomaviruses as antigens. Absorbance spread measurements are shown as blue dots, representing the German (left) and Ivorian panels (right), respectively. The COV is shown as dashed line (values are given in legend of Figure 3). Solid line within the graph: age trendline. (TIF) [file ppat.1003429.s009.tif]
