# Supplementary material for: Novel Polyomaviruses of Nonhuman Primates: Genetic and Serological Predictors for the Existence of Multiple Unknown Polyomaviruses within the Human Population
Source: PLoS Pathog. 2013 Jun 20;9(6):e1003429. doi: 10.1371/journal.ppat.1003429 (PMC3688531; doi:10.1371/journal.ppat.1003429)
Supplement: Table S1 — Primate species and tissues tested with generic polyomavirus PCR. (DOC) [file ppat.1003429.s010.doc]

**Table S1. Primate species and tissues tested with generic polyomavirus PCR.**

| **Primate species** | **Number of animals** | **Tissue analyzed** | **% of positive animals** |
| --- | --- | --- | --- |
| **HAPLORRHINI** |  |  |  |
| **Catarrhini** |  |  |  |
| **Family: Cercopithecidae** |  |  |  |
| **Subfamily: Cercopithecinae** |  |  |  |
| ***Genus: Cercocebus*** |  |  |  |
| *Cercocebus atys* | 1 | Kidney | 0 |
| ***Genus: Cercopithecus*** |  |  |  |
| *Cercopithecus diana* | 2 | Blood, kidney, lung, spleen | 0 |
| *Cercopithecus erythrotis* | 1 | Intestine, spleen | 100 |
| *Cercopithecus hamlyni* | 1 | Kidney | 0 |
| ***Genus: Erythrocebus*** |  |  |  |
| *Erythrocebus patas* | 2 | Blood, kidney, lung, spleen | 0 |
| ***Genus: Macaca*** |  |  |  |
| *Macaca fascicularis* | 16 | Blood, lung, lymph node, spleen | 13 |
| *Macaca fuscata* | 2 | Blood, kidney, lung, spleen | 0 |
| *Macaca maura* | 1 | Blood | 0 |
| *Macaca mulatta* | 35 | Blood, kidney, lung, skin, spleen | 0 |
| *Macaca silenus* | 6 | Blood | 0 |
| *Macaca sylvanus* | 1 | Liver, lung, spleen | 0 |
| *Macaca thibetana* | 2 | Blood | 0 |
| ***Genus: Mandrillus*** |  |  |  |
| *Mandrillus sphinx* | 4 | Intestine, kidney, lung, lymph node, spleen | 0 |
| ***Genus: Papio*** |  |  |  |
| *Papio hamadryas* | 2 | Kidney, lung, spleen | 0 |
| **Subfamily: Colobinae** |  |  |  |
| ***Genus: Colobus*** |  |  |  |
| *Colobus guereza* | 14 | Blood, heart, liver, lung, lymph node, spleen, stomach | 0 |
| *Colobus polykomos* | 6 | CMR^a^, feces, intestine, muscle | 0 |
| ***Genus: Piliocolobus*** |  |  |  |
| *Piliocolobus badius* | 57 | Blood, CMR, feces, intestine, liver, lung, lymph node, muscle, spleen, stomach | 9 |
| *Piliocolobus rufomitratus* | 1 | Spleen | 100 |

**… Table S1 continued**

| **Primate species** | **Number of animals** | **Tissue analyzed** | **% of positive animals** | |
| --- | --- | --- | --- | --- |
| ***Genus: Semnopithecus*** |  |  | |  |
| *Semnopithecus entellus* | 2 | Blood, spleen | 0 | |
| **Family: Hominidae** |  |  |  | |
| ***Genus: Gorilla*** |  |  |  | |
| *Gorilla beringei* | 1 | Feces | 100 | |
| *Gorilla gorilla* | 31 | Blood, feces, intestine, lung, lymph node, skin, spleen, urine | 6 | |
| ***Genus: Pan*** |  |  |  | |
| *Pan paniscus* | 7 | Blood, feces | 0 | |
| *Pan troglodytes* | 115 | Blood, feces, heart, intestine, kidney, muscle, liver, lung, lymph node, skin, spleen, stomach, thymus, urine | 13 | |
| ***Genus: Pongo*** |  |  |  | |
| *Pongo pygmaeus* | 15 | Blood, feces, heart, intestine, liver, lung, lymph node, spleen, stomach, urine | 7 | |
| ***Genus: Symphalangus*** |  |  |  | |
| *Symphalangus syndactylus* | 1 | Kidney, lung | 0 | |
| **Platyrrhini** |  |  |  | |
| ***Genus: Ateles*** |  |  |  | |
| *Ateles paniscus* | 3 | Blood, heart, intestine, kidney, liver, lung, skin, spleen, stomach, urine | 33 | |
| ***Genus: Callicebus*** |  |  |  | |
| *Callicebus cupreus* | 2 | Intestine, kidney, lung, spleen | 0 | |
| ***Genus: Callimico*** |  |  |  | |
| *Callimico goeldii* | 1 | Heart, kidney, liver, lung, lymph node | 0 | |
| ***Genus: Callithrix*** |  |  |  | |
| *Callithrix jacchus* | 20 | Intestine, kidney, lung, lymph node, spleen | 0 | |
| *Callithrix penicillata* | 1 | Blood, kidney, spleen | 0 | |
| ***Genus: Cebus*** |  |  |  | |
| *Cebus albifrons* | 3 | Kidney, lung, lymph node, skin, spleen | 67 | |
| *Cebuella pygmaea* | 1 | Heart, kidney, liver, lung, lymph node, spleen | 0 | |
| ***Genus: Leontopithecus*** |  |  |  | |
| *Leontopithecus rosalia* | 3 | Kidney, lung, lymph node, spleen | 0 | |
| ***Genus: Pithecia*** |  |  |  | |
| *Pithecia pithecia* | 3 | Blood, kidney, liver, lung, lymph node, spleen | 67 | |

**… Table S1 continued**

| **Primate species** | **Number**  **of animals** | **Tissue analyzed** | **% of positive**  **animals** |
| --- | --- | --- | --- |
| ***Genus: Saguinus*** |  |  |  |
| *Saguinus fuscicollis* | 3 | Kidney, lung, lymph node, spleen | 0 |
| *Saguinus labiatus* | 5 | Blood, heart, intestine, kidney, liver, lung, lymph node, spleen, thymus | 0 |
| *Saguinus midas* | 1 | Kidney, lung, lymph node, spleen | 0 |
| *Saguinus oedipus* | 5 | Intestine, lung, lymph node, spleen, stomach | 0 |
| *Saimiri sciureus* | 18 | Blood, intestine, kidney, lung, lymph node, spleen | 11 |
| **STREPSIRRHINI** |  |  |  |
| **Family: Lemuridae** |  |  |  |
| ***Genus: Eulemur*** |  |  |  |
| *Eulemur fulvus* | 1 | Kidney, spleen | 0 |
| ***Genus: Lemur*** |  |  |  |
| *Lemur catta* | 1 | Intestine, kidney, lung, lymph node, spleen | 0 |
| ***Genus: Varecia*** |  |  |  |
| *Varecia variegata* | 1 | Kidney, lymph node, spleen | 0 |
| **Family: Lorisidae** |  |  |  |
| ***Genus: Nycticebus*** |  |  |  |
| *Nycticebus coucang* | 1 | Blood, skin | 0 |
| ***Genus: Perodicticus*** |  |  |  |
| *Perodicticus potto* | 1 | Liver, spleen | 0 |

^a^ CMR: muscle or other tissues from red colobus monkeys partly consumed by chimpanzees.
