# Supplementary material for: Novel Polyomaviruses of Nonhuman Primates: Genetic and Serological Predictors for the Existence of Multiple Unknown Polyomaviruses within the Human Population
Source: PLoS Pathog. 2013 Jun 20;9(6):e1003429. doi: 10.1371/journal.ppat.1003429 (PMC3688531; doi:10.1371/journal.ppat.1003429)
Supplement: Table S2 — Primers used for amplification of nonhuman primate polyomaviruses. (DOC) [file ppat.1003429.s011.doc]

**Table S2. Primers used for amplification of nonhuman primate polyomaviruses.**

| **Primer** | **PCR round** | **Target** | **Sequence (5´-3´)** | **Fragment size (bp)** | **Annealing temperature (°C)** |
| --- | --- | --- | --- | --- | --- |
| **Generic PCR-1** |  |  |  |  |  |
| VP1-1 sense | 1 | PyV VP1 | CCAGACCCAACTARRAATGARAA | 800-1100 | 64 |
| VP1-1 antisense |  |  | AACAAGAGAACACAAAT(n/i)TTTCC(n/i)CC**^a^** |  |  |
| VP1-2 sense | 2 |  | ATGAAAATGGGGTTGGCCC(n/i)CT(n/i)TGYAARG | 250-270 | 50 |
| VP1-2 antisense |  |  | CCCTCATAAACCCGAACYTCYTC(h/i)ACYTG |  |  |
| **Generic PCR-2** |  |  |  |  |  |
| VP1-1 sense | 1 | PyV VP1 | CCTGATCCTTCTA(r/i)(r/i)AATGA(r/i)AA | 800-1100 | 46 |
| VP1-1 antisense |  |  | AATAAGAAGCATCAGAT(n/i)TTyCC(n/i)CC |  |  |
| VP1-2 sense | 2 |  | ATGAGAATGGAGTGGGCCC(n/i)CT(n/i)TG(y/i)AA(r/i)G | 250-270 | 50 |
| VP1-2 antisense |  |  | CCCTCATATATTCTAACYTCYTC(h/i)AC(y/i)TG |  |  |
| **Long-distance PCRs** |  |  |  |  |  |
| PtrovPyV3 sense | 1 | PtrovPyV3 VP1 | ACCCTGTAACTTCTCTTTTGTCT | 5287 | 63 |
| PtrovPyV3 antisense |  |  | AGGGAGGCCTCTATACTGCTTGT |  |  |
| PtrovPyV3 sense | 2 |  | TCTCTTTTCAATAGTCTAATGCCTAA | 5249 | 60 |
| PtrovPyV3 antisense |  |  | CTGCTTGTGCCCATCATTGT |  |  |
| PtrovPyV3 sense | 3 |  | TAAAGGGGCAACCCATGGAGG | 5193 | 62 |
| PtrovPyV3 antisense |  |  | AAGCCACAAATATCTGCACAGC |  |  |
| PtrovPyV4 sense | 1 | PtrovPyV4 VP1 | AACCCCTACCCTGTGACTTC | 5265 | 60 |
| PtrovPyV4 antisense |  |  | CCCACAAATGTCTGCACAGC |  |  |
| PtrovPyV4 sense | 2 |  | CTGTGACTTCACTTTTGACTTCT | 5253 | 59 |
| PtrovPyV4 antisense |  |  | CACAAATGTCTGCACAGCTC |  |  |

**… Table S2** continued

| **Primer** | **PCR round** | **Target** | **Sequence (5´-3´)** | **Fragment size (bp)** | **Annealing temperature (°C)** |
| --- | --- | --- | --- | --- | --- |
| PtrovPyV5 sense | 1 | PtrovPyV5 VP1 | ACCCATATCCAGTGAGTTCTCTCC | 4968 | 64 |
| PtrovPyV5 antisense |  |  | ACATTAAAATATCTGGGTAGGCCTCT |  |  |
| PtrovPyV5 sense | 2 |  | AATAGTTTGTTTTCTGGTCTTATGCC | 4923 | 61 |
| PtrovPyV5 antisense |  |  | GGCCTCTCCAGTTTTGGG |  |  |
| PtrovPyV5 sense | 3 |  | TTCAGGGACAACCCATGGAA | 4880 | 60 |
| PtrovPyV5 antisense |  |  | TTTGGGACTCTGAATAATTAGTATGT |  |  |
| PtrosPyV2 sense | 1 | PtrosPyV2 VP1 | ACCCATATCCAGTTAGCATGTTG | 4933 | 61 |
| PtrosPyV2 antisense |  |  | TCTAGGTAGGCCTCTCCAGTT |  |  |
| PtrosPyV2 sense | 2 |  | AGCATGTTGCTAAACAGTGTATTT | 4909 | 61 |
| PtrosPyV2 antisense |  |  | CCTCTCCAGTTTTGGGAATTACTAT |  |  |
| PtrosPyV2 sense | 3 |  | AACAGTGTATTTTCCAATCTTATGCC | 4865 | 61 |
| PtrosPyV2 antisense |  |  | ATGCATCCCCACAATATCTGC |  |  |
| PrufPyV1 sense | 1 | PrufPyV1 VP1 | AGAGCTGTCAAAAACCCCTACCCA | 5113 | 64 |
| PrufPyV1 antisense |  |  | AGGTAGGCCCCTGTACCTGGC |  |  |
| PrufPyV1 sense | 2 |  | AGCTTGCTTGGAAGCCTGTTT | 5069 | 60 |
| PrufPyV1 antisense |  |  | ACCTGGCTGCTCCATTGTCT |  |  |
| PrufPyV1 sense | 3 |  | TGCCTAAAATGCAAGGGCAACCT | 5010 | 64 |
| PrufPyV1 antisense |  |  | CAGGATTCCACAAATATCAGCACATGA |  |  |
| CeryPyV1 sense | 1 | CeryPyV1 VP1 | AGGACAGTGAGAAACCCATACCCA | 5162 | 64 |
| CeryPyV1 antisense |  |  | TGACAGTCCCCTCCACTGCT |  |  |
| CeryPyV1 sense | 2 |  | AAGGGTGGATGGGCAACCTA | 5078 | 60 |
| CeryPyV1 antisense |  |  | TGGGTCCCTGAACTGTTGGT |  |  |

**… Table S2 continued**

| **Primer** | **PCR round** | **Target** | **Sequence (5´-3´)** | **Fragment size (bp)** | **Annealing temperature (°C)** |
| --- | --- | --- | --- | --- | --- |
| MfasPyV1 sense | 1 | MfasPyV1 VP1 | TGCTAAATAGCTTGTTCTCTGGTCT | 5042 | 64 |
| MfasPyV1 antisense |  |  | GCAAAGAGCTCACTGGGTATGGA |  |  |
| MfasPyV1 sense | 2 |  | TGTTCTCTGGTCTTATGCCACC | 5022 | 61 |
| MfasPyV1 antisense |  |  | CACTGGGTATGGATTTTTAACAGC |  |  |
| ApanPyV1 sense | 1 | ApanPyV1 VP1 | CTCCACCCTTCTGAACAATC | 5212 | 58 |
| ApanPyV1 antisense |  |  | CCCTGTAAGCCATTGTTTCT |  |  |
| ApanPyV1 sense | 2 |  | GAACAATCTGTTCACTGGCT | 5188 | 56 |
| ApanPyV1 antisense |  |  | TTGTTTCTGCAGCATTTGTG |  |  |
| ApanPyV1 sense | 3 |  | GTTCACTGGCTATTTGCCTA | 5162 | 57 |
| ApanPyV1 antisense |  |  | GTGTGAAAGCCAACAATGTC |  |  |
| CalbPyV1 sense | 1 | CalbPyV1 VP1 | AGAAAGAGACTTGTGAGGAATCCA | 4994 | 61 |
| CalbPyV1 antisense |  |  | TCTGGGAAAACCCCTGAATCT |  |  |
| CalbPyV1 sense | 2 |  | TGTGAGGAATCCATACCCTGTCA | 4960 | 61 |
| CalbPyV1 antisense |  |  | ACTGCCCATCAGGTTGTACT |  |  |
| SsciPyV1 sense | 1 | SsciPyV1 VP1 | AGTACAGAGGGCTGCCTAGA | 5067 | 62 |
| SsciPyV1 anti sense |  |  | GCAAAGTGCTTACAGGGTATGGA |  |  |
| SsciPyV1 sense | 2 |  | GGCTGCCTAGATACTTTAAGCTTGT | 5030 | 61 |
| SsciPyV1 anti sense |  |  | AGGCAGCCCTCTGTACTTAT |  |  |

**^a^** (n/i), a;c:g:t:inosine in equal percentages
