# Supplementary material for: Novel Polyomaviruses of Nonhuman Primates: Genetic and Serological Predictors for the Existence of Multiple Unknown Polyomaviruses within the Human Population
Source: PLoS Pathog. 2013 Jun 20;9(6):e1003429. doi: 10.1371/journal.ppat.1003429 (PMC3688531; doi:10.1371/journal.ppat.1003429)
Supplement: Table S3 — Known and novel polyomaviruses used in phylogenetic analysis. (DOC) [file ppat.1003429.s012.doc]

**Table S3. Known and novel polyomaviruses used in phylogenetic analysis.**

| **Virus species** | **Virus abbreviation** | **Genome or partial VP1 gene (bp)** | **Host species** | **GenBank accession number** |
| --- | --- | --- | --- | --- |
| Ateles paniscus polyomavirus 1 | ApanPyV1 | 5273 | Red-faced spider monkey | JX159987 |
| Avian polyomavirus | APyV^a^ | 4981 | Parrot and other bird species | NC_004764 |
| Baboon polyomavirus 1 | SA12 | 5230 | Chacma baboon; vervet monkey | [NC_007611](http://www.ncbi.nlm.nih.gov/sites/entrez?Db=genome&Cmd=ShowDetailView&TermToSearch=23927) |
| Bat polyomavirus 1 | BatPyV1 | 5081 | North American Bat | [FJ188392](http://www.ncbi.nlm.nih.gov/sites/entrez?Db=genome&Cmd=ShowDetailView&TermToSearch=22960) |
| Bat polyomavirus 2a | BatPyV2a | 5201 | South American Bat | JQ958892 |
| Bat polyomavirus 2b | BatPyV2b | 5041 | .. | JQ958891 |
| Bat polyomavirus 2c | BatPyV2c | 5187 | .. | JQ958890 |
| Bat polyomavirus 3a1 | BatPyV3a1 | 5019 | .. | JQ958886 |
| Bat polyomavirus 3a2 | BatPyV3a2 | 5058 | .. | JQ958888 |
| Bat polyomavirus 3b | BatPyV3b | 4903 | .. | JQ958893 |
| Bat polyomavirus 4a | BatPyV4a | 5371 | .. | JQ958887 |
| Bat polyomavirus 4b | BatPyV4b | 5352 | .. | JQ958889 |
| B-lymphotropic polyomavirus | LPyV | 5270 | African green monkey | [K02562](http://www.ncbi.nlm.nih.gov/sites/entrez?Db=genome&Cmd=ShowDetailView&TermToSearch=17050) |
| BK polyomavirus | BKPyV | 5089 | Human | M23122 |
| Bornean orangutan polyomavirus | OraPyV1 | 5168 | Orangutan | FN356900 |
| Bovine polyomavirus | BoPyV | 4697 | Cattle | [NC_001442](http://www.ncbi.nlm.nih.gov/sites/entrez?Db=genome&Cmd=ShowDetailView&TermToSearch=10168) |
| Canary polyomavirus | CaPyV | 5421 | Canary bird | GU_345044 |
| Cebus albifrons polyomavirus 1 | CalbPyV1 | 5012 | White-fronted capuchin | JX159988 |
| Cebus albifrons polyomavirus 2 | CalbPyV2 | 203 ^b^ | .. | JX159997 |
| Cebus albifrons polyomavirus 3 | CalbPyV3 | 212 ^b^ | .. | JX159998 |
| Cercopithecus erythrotis polyomavirus 1 | CeryPyV1 | 5189 | Red-eared guenon | JX159985 |
| Crow polyomavirus | CPyV | 5079 | Jackdaw | DQ192570 |
| Chimpanzee polyomavirus | ChPyV | 5063 | Central Chimpanzee | FR692335 |
| Horse polyomavirus | EPyV | 4987 | Horse | JQ412134 |
| Finch polyomavirus | FPyV | 5278 | Bullfinch | DQ192571 |
| Gorilla beringei graueri polyomavirus 1 | GbergPyV1 | 212 ^b^ | Eastern lowland gorilla | JX159992 |
| Gorilla gorilla gorilla polyomavirus 1 | GgorgPyV1^c^ | 5300 | Western lowland gorilla | HQ385752 |
| Gorilla gorilla gorilla polyomavirus 2 | GgorgPyV2 | 212 ^b^ | .. | JX159993 |
| Goose hemorrhagic polyomavirus | GHPyV | 5256 | Goose | AY140894 |
| Hamster polyomavirus | HaPyV | 5366 | Hamster | NC_001663; AJ006015 |
| Human polyomavirus 6 | HPyV6 | 4926 | Human | HM011558 |

**… Table S3 continued**

| **Virus species** | **Virus abbreviation** | **Genome or partial VP1 gene (bp)** | | **Host species** | | **GenBank accession number** | |
| --- | --- | --- | --- | --- | --- | --- | --- |
| Human polyomavirus 7 | HPyV7 | 4949 | | .. | HM011565 | |  |
| Human polyomavirus 9 | HPyV9 | 5026 | | .. | HQ696595 | |  |
| Human polyomavirus 10 | HPyV10 | 4939 | | .. | JX262162 | |  |
| Human polyomavirus 12 | HPyV12 | 5033 | | .. | JX308829 | |  |
| JC polyomavirus | JCPyV | 5147 | | .. | JF424942 | |  |
| KI polyomavirus | KIPyV | 5040 | .. | | NC_009238 | |  |
| Macaca fascicularis polyomavirus 1 | MfasPyV1 | 5087 | Crab-eating macaque | | JX159986 | |  |
| Mastomys polyomavirus | MasPyV | 4899 | Mouse | | AB_588640 | |  |
| Merkel cell polyomavirus | MCPyV | 5387 | Human | | JF813003 | |  |
| Murine pneumotropic polyomavirus | MPtV | 4754 | Mouse | | EF186666 | |  |
| Murine polyomavirus | MPyV | 5380 | .. | | U27812 | |  |
| MW polyomavirus | MWPyV | 4927 | Human | | JQ898291 | |  |
| Pan troglodytes schweinfurthii polyomavirus 2 | PtrosPyV2 | 4970 | Eastern Chimpanzee | | JX159983 | |  |
| Pan troglodytes troglodytes polyomavirus 1 | PtrotPyV1 | 212 ^b^ | Central Chimpanzee | | JX159991 | |  |
| Pan troglodytes verus polyomavirus 1a | PtrovPyV1a^c^ | 5303 | Western Chimpanzee | | HQ385746 | |  |
| Pan troglodytes verus polyomavirus 1b | PtrovPyV1b^c^ | 5301 | .. | | HQ385747 | |  |
| Pan troglodytes verus polyomavirus 2a | PtrovPyV2a^c^ | 5309 | .. | | HQ385748 | |  |
| Pan troglodytes verus polyomavirus 2c | PtrovPyV2c^c^ | 5315 | .. | | HQ385749 | |  |
| Pan troglodytes verus polyomavirus 3 | PtrovPyV3 | 5333 | .. | | JX159980 | |  |
| Pan troglodytes verus polyomavirus 4 | PtrovPyV4 | 5349 | .. | | JX159981 | |  |
| Pan troglodytes verus polyomavirus 5 | PtrovPyV5 | 4994 | .. | | JX159982 | |  |
| Pan troglodytes verus polyomavirus 6 | PtrovPyV6 | 212 ^b^ | .. | | JX159990 | |  |
| Piliocolobus badius polyomavirus 1 | PbadPyV1 | 212 ^b^ | Western red colobus | | JX159995 | |  |
| Piliocolobus badius polyomavirus 2 | PbadPyV2 | 203 ^b^ | .. | | JX159996 | |  |
| Piliocolobus rufomitratus polyomavirus 1 | PrufPyV1 | 5140 | Eastern red colobus | | JX159984 | |  |
| Pithecia pithecia polyomavirus 1 | PpitPyV1 | 205 ^b^ | White-faced saki | | JX159999 | |  |
| Pongo pygmaeus polyomavirus 1 | PpygPyV1 | 215 ^b^ | Orangutan | | JX159994 | |  |
| Raccoon polyomavirus | RacPyV | 5016 | Raccoon | | JQ178241 | |  |
| Saimiri sciureus polyomavirus 1 | SsciPyV1 | 5067 | Squirrel monkey | | JX159989 | |  |
| Simian virus 40 | SV40 | 5162 | Rhesus monkey | | EF579662 | |  |

**… Table S3 continued**

| **Virus species** | **Virus abbreviation** | **Genome or partial VP1 gene (bp)** | | **Host species** | | **GenBank accession number** | |
| --- | --- | --- | --- | --- | --- | --- | --- |
| Sea lion polyomavirus 1 | SLPyV | 5112 | Sea lion | | GQ331138 | |  |
| Squirrel monkey polyomavirus | SqPyV | 5075 | Squirrel monkey | | [NC_009951](http://www.ncbi.nlm.nih.gov/sites/entrez?Db=genome&Cmd=ShowDetailView&TermToSearch=21537) | |  |
| STL polyomavirus | STLPyV | 4776 | | Human | NC_020106 | |  |
| Sumatran orangutan polyomavirus | OraPyV2 | 5358 | | Orangutan | FN356901 | |  |
| Trichodysplasia spinulosa-associated polyomavirus | TSPyV | 5232 | | Human | [NC_014361](http://www.ncbi.nlm.nih.gov/sites/entrez?Db=genome&Cmd=ShowDetailView&TermToSearch=21537) | |  |
| WU polyomavirus | WUPyV | 5229 | | .. | GU296381 | |  |

^a^ Former name: BFDPyV.

^b^  Partial VP1 sequence.

^c^ Abbreviation in original reference (Leendertz et al., 2011): GggPyV1 (GgorgPyV1), PtsPyV1 (PtrosPyV1), PtvPyV1a (PtrovPyV1a), PtvPyV1b (PtrovPyV1b), PtvPyV2a (PtrovPyV2a), PtvPyV2b (PtrovPyV2b), PtvPyV2c (PtrovPyV2c).
