# Supplementary material for: Novel Polyomaviruses of Nonhuman Primates: Genetic and Serological Predictors for the Existence of Multiple Unknown Polyomaviruses within the Human Population
Source: PLoS Pathog. 2013 Jun 20;9(6):e1003429. doi: 10.1371/journal.ppat.1003429 (PMC3688531; doi:10.1371/journal.ppat.1003429)
Supplement: Table S4 — Genomes and encoded proteins of the novel nonhuman primate polyomaviruses. (DOC) [file ppat.1003429.s013.doc]

**Table S4. Genomes and encoded proteins of the novel nonhuman primate polyomaviruses.**

|  | | **VP1** | | **VP2** | | | **Large T** | | **Small T** | | **Agnoprotein** | |
| --- | --- | --- | --- | --- | --- | --- | --- | --- | --- | --- | --- | --- |
| **Novel polyomavirus** | **Genome size bp** | **Coding region** | **aa** | **Coding region** | **aa** | **Coding region** | | **aa** | **Coding region** | **aa** | **Coding region** | **aa** |
| ApanPyV1 | 5273 | 1581-2714 | 377 | 529-1470 | 313 | 5273-4750^a^ 4549-2767^b^ | | 768 | 5273-4680 | 197 | - | - |
| CalbPyV1 | 5012 | 1656-2717 | 353 | 731-1693 | 320 | 5012-4776 4358-2757 | | 612 | 5012-4521 | 163 | - | - |
| CeryPyV1 | 5189 | 1576-2670 | 364 | 636-1697 | 353 | 5189-4945 4598-2753 | | 696 | 5189-4671 | 172 | 391-603 | 70 |
| MfasPyV1 | 5087 | 1450-2565 | 371 | 516-1568 | 350 | 5087-4853 4492-2628 | | 699 | 5087-4518 | 189 | - | - |
| PrufPyV1 | 5140 | 1045-2553 | 502 | 390-1088 | 232 | 5140-4925 4266-2596 | | 628 | 5140-4562 | 192 | - | - |
| PtrovPyV3 | 5333 | 1497-2660 | 387 | 575-1531 | 318 | 5333-5099 4672-2715 | | 730 | 5333-4746 | 195 | - | - |
| PtrovPyV4 | 5349 | 1515-2666 | 383 | 596-1549 | 317 | 5349-5118 4688-2719 | | 733 | 5349-4768 | 193 | - | - |
| PtrovPyV5 | 4994 | 1436-2551 | 371 | 493-1554 | 353 | 4994-4760 4400-2614 | | 673 | 4994-4425 | 189 | - | - |
| PtrosPyV2 | 4970 | 1432-2538 | 368 | 504-1553 | 349 | 4970-4733 4374-2615 | | 665 | 4970-4401 | 189 | - | - |
| SsciPyV1 | 5067 | 1707-2780 | 357 | 767-1765 | 332 | 5067-4833 4556-2818 | | 657 | 5067-4576 | 163 | - | - |

^a^ Exon 1; ^b^ Exon 2
