# Supplementary material for: Novel Polyomaviruses of Nonhuman Primates: Genetic and Serological Predictors for the Existence of Multiple Unknown Polyomaviruses within the Human Population
Source: PLoS Pathog. 2013 Jun 20;9(6):e1003429. doi: 10.1371/journal.ppat.1003429 (PMC3688531; doi:10.1371/journal.ppat.1003429)
Supplement: Table S5 — Putative functional motifs in the large T-antigens of the novel NHP polyomaviruses. (DOCX) [file ppat.1003429.s014.docx]

**Table S10.** **Putative functional motifs in the large T-antigens of the novel NHP polyomaviruses.**

|  | **CR1**  **LXXLL** | **DnaJ**  **HPDKGG** | **Bub-1**  **W^D^/_E_AWW** | **CUL-7**  **FNEEN** | **pRb1**  **LXCXE** | **Zn-finger**  **CX_2_CX_7_HX_3_H** | **ATPase**  **GPX_3_GKT and GX_3_VNLE** | **NLS**  **PKKKRKV** | **HRD^b^** |
| --- | --- | --- | --- | --- | --- | --- | --- | --- | --- |
| ApanPyV1 | LMDLL  (13-17)**^a^** | HPDKGG  (42-47) | absent | FNTEW  (177-181) | LFCNE  (193-197) | **C**TK**C**AKKILKA**H**YNY**H**  (417-432) | GPINSGKT and GSIKVNLE  (538-545) and (615-622) | PKPKKSK  (243-249) | no |
| CalbPyV1 | LMELL  (13-17) | HPDKGG  (42-47) | absent | absent | absent | **C**EK**C**DAKLIPD**H**YKY**H**  (262-278) | GPVNTGKT and GAVKVNLE  (98-105) and (466-473) | TKKKKTS  (88-94) | no |
| CeryPyV1 | LMDLL  (13-17) | HPDKGG  (42-47) | WEAWW  (90-94) | FNPEE  (259-263) | LFCHE  (105-109) | **C**RK**C**QKKDQPY**H**FKY**H**  (204-219) | GPIDSGKT and GSVKVNLE  (428-435) and (505-512) | PKKKRKV  (128-134) | yes  (C-terminal) |
| MfasPyV1 | LMDLL  (13-17) | HPDKGG  (42-47) | WGKWW  (109-113) | absent | LFCTE  (125-129) | **C**SK**C**EKKQHKFHYNY**H** | GPINSGKT and GTIKVNLE  (491-498) and (568-575) | PKRRKSD  (191-197) | no |
| PtrovPyV3 | LISLL  (13-17) | HPDKGG  (42-47) | absent | absent | absent | **C**AK**C**EKKQLKV**H**YMF**H**  (393-408) | GPVNTGKT and GAVKVNLE  (229-236) and (589-596) | FKRRRKT  (141-147) | no |
| PtrovPyV4 | LIALL  (13-17) | HPDKGG  (42-47) | absent | FNTEL  (62-66)  FNESY  (386-390) | absent | **C**KK**C**DKKQFKV**H**YMF**H**  (392-407) | GPVNTGKT and GAVKVNLE  (229-236) and (589-596) | FKRRRKT  (140-146) | no |
| PtrovPyV5 | LMDLL  (13-17) | HPDKGG  (42-47) | WGKWW  (107-111) | absent | LFCSE  (123-127) | **C**AK**C**EKKSHKF**H**YNY**H**  (343-358) | GPINSGKT and GTIKVNLE  (465-472) and (542-549) | PKRKKTE  (164-170) | no |
| PtrosPyV2 | YMELL  (13-17) | HPDKGG  (42-47) | absent | absent | LYCSE  (108-112) | **C**DK**C**AKKKNKF**H**YNY**H**  (336-351) | GPLNCGKT and GTIKVNLE  (458-465) and (535-542) | PPKKRKV  (159-165) | no |
| PrufPyV1 | LMELL  (13-17) | HPDKGG  (42-47) | absent | absent | absent | **C**NK**C**DSLKTKA**H**EY**H^c^**  (298-312) | GPVNSGKT and GAVPVNLE  (417-424) and (444-501) | PEKRRRS  (95-101) | no |
| SsciPyV1 | LMELL  (13-17) | HPDKGG  (42-47) | WERWW  (88-92) | FNEDF  (95-99) | LFCHE  (101-105) | **C**CK**C**DQKVVVD**H**YKY**H**  (310-325) | GPVNTGKT and GAVKVNLE  (436-443) and (512-519) | PKKKKTP  (135-141) | no |

^a^ The numbers in parenthesis refer to the amino acid residues

^b^ CX_2_CX_7_HX_2_H

^c^ host range domain
