# Supplementary material for: Novel Polyomaviruses of Nonhuman Primates: Genetic and Serological Predictors for the Existence of Multiple Unknown Polyomaviruses within the Human Population
Source: PLoS Pathog. 2013 Jun 20;9(6):e1003429. doi: 10.1371/journal.ppat.1003429 (PMC3688531; doi:10.1371/journal.ppat.1003429)
Supplement: Table S6 — Correlation of seroreactivities against VP1 antigens of polyomaviruses. (DOC) [file ppat.1003429.s015.doc]

**Table S17.** **Correlation of seroreactivities against VP1 antigens of polyomaviruses.**

|  | **Correlation coefficient (r)^*^** | | | | | | | | | | | | |  | |  | | | |
| --- | --- | --- | --- | --- | --- | --- | --- | --- | --- | --- | --- | --- | --- | --- | --- | --- | --- | --- | --- |
|  | **ChPyV** | | **PtrovPyV3** | | **PtrovPyV4** | | | **PtrosPyV2** | | **HPyV9** | | **JCPyV** | | | | |  |  | |
|  | German | Ivorian | German | Ivorian | | German | Ivorian | German | Ivorian | German | Ivorian | | German | | Ivorian | |  | |  |
| ChPyV | 1 | 1 | 0.450 | 0.362 | | 0.535 | 0.307 | 0.517 | 0.395 | 0.406 | 0.295 | | 0.207 | | 0.289 | |  | |  |
| PtrovPyV3 | 0.450 | 0.362 | 1 | 1 | | 0.620 | 0.452 | 0.424 | 0.328 | 0.465 | 0.263 | | 0.196 | | 0.178 | |  | |  |
| PtrovPyV4 | 0.535 | 0.307 | 0.620 | 0.452 | | 1 | 1 | 0.490 | 0.411 | 0.471 | 0.243 | | 0.284 | | 0.250 | |  | |  |
| PtrosPyV2 | 0.517 | 0.395 | 0.424 | 0.328 | | 0.490 | 0.411 | 1 | 1 | 0.599 | 0.411 | | 0.398 | | 0.294 | |  | |  |
| HPyV9 | 0.406 | 0.295 | 0.465 | 0.263 | | 0.471 | 0.243 | 0.599 | 0.411 | 1 | 1 | | 0.460 | | 0.368 | |  | |  |
| JCPyV | 0.207 | 0.289 | 0.196 | 0.178 | | 0.284 | 0.250 | 0.398 | 0.294 | 0.460 | 0.368 | | 1 | | 1 | |  | |  |

^*^ Correlation analyses were performed with the Spearman rank correlation test.
