# Supplementary material for: Novel Polyomaviruses of Nonhuman Primates: Genetic and Serological Predictors for the Existence of Multiple Unknown Polyomaviruses within the Human Population
Source: PLoS Pathog. 2013 Jun 20;9(6):e1003429. doi: 10.1371/journal.ppat.1003429 (PMC3688531; doi:10.1371/journal.ppat.1003429)
Supplement: Text S1 — LT-ag binding motifs in NCCR of novel NHP polyomaviruses. (DOCX) [file ppat.1003429.s016.docx]

**Text S5. LT-ag binding motifs in NCCR of novel NHP polyomaviruses.**

The NCCRs of all NHP viruses described in this study possess one or several potential LTag binding motifs (GAGGC [[1](#_ENREF_1)]; **Figure S6**). The MfasPyV1, PrufPyV1 and PtrosPyV2 NCCRs contain one LTag binding motif, while two are present in the NCCRs of PtrovPyV3and PtrovPyV5. Three putative LTag binding sites are present in the NCCRs of CalbPyV1, CeryPyV1and PtrovPyV4, while four can be detected in the ApanPyV1 and SsciPyV1 NCCRs. For the NCCRs that contain multiple LTag binding motifs, the motifs are scattered. Only in ApanPyV1, CalbPyV1 and SsciPyV1 are 2 motifs direct repeats. AT-rich regions are found in all NCCRs, but inverted repeats are rare. In fact, only the MfasPyV1 NCCR contains the inverted repeats TAAGTTGCTA and TAGCAACTA. Interestingly, the NCCRs of MfasPyV1 and PtrovPyV5 share 87% nucleotide identity in a region of 146 nucleotides, whereas the NCCRs of PtrosPyV2 and PtrovPyV5 share 84% nucleotide identity in a region of 135 nucleotides (**Figure S7)**. No significant identity between the other NCCRs exists.

**References**

1. Pomerantz BJ, Hassell JA (1984) Polyomavirus and simian virus 40 large T antigens bind to common DNA sequences. J Virol 49: 925-937.
