# Supplementary material for: Novel Polyomaviruses of Nonhuman Primates: Genetic and Serological Predictors for the Existence of Multiple Unknown Polyomaviruses within the Human Population
Source: PLoS Pathog. 2013 Jun 20;9(6):e1003429. doi: 10.1371/journal.ppat.1003429 (PMC3688531; doi:10.1371/journal.ppat.1003429)
Supplement: Text S2 — Motifs in large T antigens of novel NHP polyomaviruse. (DOCX) [file ppat.1003429.s017.docx]

**Text S8. Motifs in large T antigens of novel NHP polyomaviruses**.

The LTag of all novel NHP described here contain several motifs similar to previously described functional motifs in SV40 LTag (**Table S10 and Figure S11**) [[1](#_ENREF_1)]. They possess conserved the CR1 LXXLL motif (except PtrosPyV2 which has YXXLL), the DnaJ motif HPDKGG, the ATPase motifs GPxxxGKT and GxxxVNLE, a putative nuclear localization signal resembling the K/R-rich SV40 motif, and the Zn-finger CX_2_CX_7_HX_3_H. The latter is found as CX_2_CX_7_HX_2_H in PrufPyV1 (**Figure S9; Table S10**). The pRb pocket consensus LXCXE is absent in CalbPyV1, PtrovPyV3, PtrovPyV4, and PrufPyV1. The SV40 LTag sequence WDEAWW binds the Bub1 protein [[2](#_ENREF_2)]. A putative Bub1-like motif is found in the LTag of CeryPyV1, MfasPyV1, PtrovPyV5 and SsciPyV1. Fwb7 is a cellular protein that interacts with the C-terminal PPTPPPEPET sequence of SV40 LTag. Only the LTag of CeryPyV1 contains a reminiscent of this sequence. The SV40 LTag CUL7 binding motif FNEEN, which spans amino acids 98-102 [[3](#_ENREF_3)], is somewhat conserved in the LTags of ApanPyV1, CeryPyV1, PtrovPyV4 and SciPyV1. PtrovPyV4 LTag possesses two putative CUL7 interaction domains. The carboxy terminal part of SV40, BKPyV and JCPyV LTag contains a host range domain. This region encompasses 38 amino acids in SV40 LTag, of which 16, respectively 18 residues are identical with the host range domain of BKPyV LTag, respectively JCPyV LTag [[1](#_ENREF_1)]. The C-terminal part of CeryPyV1 seems to possess a host range domain with 22 out of 38 residues identical with the corresponding domain of SV40 LTag (**Figure S12**). Interestingly, the genomes of polyomaviruses that encode an agnoprotein (SV40, SA12, BKPyV and JCPyV) carry a carboxy-terminal host range domain. CeryPyV, the only virus in this study, whose LTag contains a presumed host range domain, is the only virus that encodes a putative agnoprotein (**Table S4**).

**References**

1. An P, Saenz Robles MT, Pipas JM (2012) Large T antigens of polyomaviruses: amazing molecular machines. Annu Rev Microbiol 66: 213-236.

2. Hein J, Boichuk S, Wu J, Cheng Y, Freire R, et al. (2009) Simian virus 40 large T antigen disrupts genome integrity and activates a DNA damage response via Bub1 binding. J Virol 83: 117-127.

3. Ali SH, Kasper JS, Arai T, DeCaprio JA (2004) Cul7/p185/p193 binding to simian virus 40 large T antigen has a role in cellular transformation. J Virol 78: 2749-2757.
